# Electronic Structure and Interfacial Hole Transfer in a Di-Rhodium Photocatalyst on a p‑Type NiO Electrode

**DOI:** 10.1021/acs.jpcc.5c05879

**Published:** 2025-10-28

**Authors:** Francesca Fasulo, Adriana Pecoraro, Ana B. Muñoz-García, Michele Pavone

**Affiliations:** † Department of Physics “Ettore Pancini”, 9307 University of Naples Federico II, Complesso Universitario Monte Sant’Angelo via Cinita 21, Naples 80126, Italy; ‡ Department of Chemical Sciences, University of Naples Federico II, Complesso Universitario Monte Sant’Angelo via Cinita 21, Naples 80126, Italy

## Abstract

Dye-sensitized photoelectrochemical cells are a promising route for solar-driven hydrogen production, using molecular dyes to generate electron–hole pairs that drive electrochemical reactions. A key challenge is achieving efficient charge transfer among the sensitizer, electrode, and catalyst. Paddlewheel dirhodium (DiRh) complexes have recently emerged as effective single-molecule photocatalysts, showing high activity when anchored to nickel oxide (NiO) electrodes for hydrogen evolution under red light. Here, we employ density functional theory (DFT) and projection-operator diabatization (POD) analysis to investigate the electronic structure of DiRh, its interaction with NiO, and the mechanisms of interfacial hole transfer. Our results show that DiRh binds strongly to NiO through stable mono- or bi- anchored configurations, with distinct ligand contributions to the charge-transfer pathway. While anchoring improves charge-separation efficiency, it has a minimal impact on the intrinsic properties of DiRh. Notably, the monoanchored s-DiRh/NiO interface exhibits stronger coupling to the NiO valence band and reduced charge recombination, making it the most favorable configuration for rapid hole injection. These findings provide atomistic insight into the structure–function relationships at dye–catalyst/electrode interfaces, offering design guidelines for next-generation photoelectrochemical systems for renewable hydrogen production. Beyond this case study, our work validates the use of a transferable hybrid-DFT/POD approach for realistic electrode–molecule systems, providing predictive atomistic insight into their interfacial electronic structure and charge-transfer characteristics.

## Graphical abstract

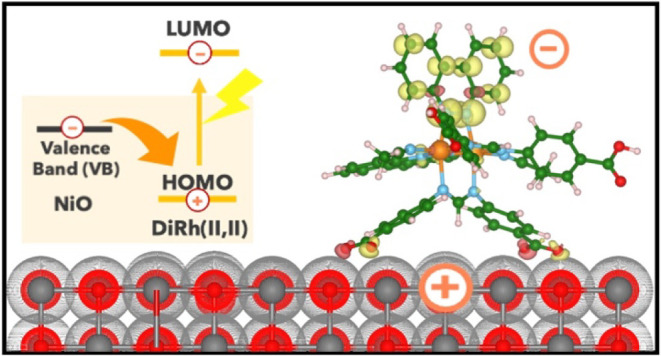

## Introduction

Dye-sensitized photoelectrochemical cells (DSPECs) are advanced devices that convert sunlight directly into chemical energy and represent a promising avenue to produce green hydrogen through water splitting.^1–4^ These cells commonly employ transition-metal oxides as electrode substrates, molecular dyes, and catalysts. Light adsorption by the dye generates electron–hole pairs, initiating subtle charge transfer processes that are critical for hydrogen and oxygen evolution reactions (HER and OER). However, these processes can be easily hampered by mismatched energy levels, kinetic barriers, or trap states, leading to charge recombination and inefficient electron/hole transport.^5–11^ Therefore, enhancing DSPEC performance calls for an efficient design of dyes, catalysts, and their interfaces. Particularly effective are single-molecule species that integrate both light-harvesting and catalytic functions, simplifying the overall cell architecture. Among these, Prof. Turro’s group explored a series of promising paddlewheel dirhodium complexes (DiRh) that stand out thanks to their dual functionality and versatility in catalyzing various reactions, including HER.^12–15^ These complexes, such as the *cis*-[Rh_2_(DPhF)_2_(bncn)_2_]­(BF_4_)_2_ (DPhF = *N*,*N*′-diphenylformamidinate, bncn = benzocinnoline), exhibit broad light absorption from ultraviolet to near-infrared, making them efficient photocatalysts in acidic solutions.^16–23^

In polar aprotic solvent (DMF), these DiRh complexes show significant absorption centered at 625 nm, which is attributed to singlet metal-to-ligand charge transfer (^1^ML-LCT). This involves electron transfer from the highest occupied molecular orbital (HOMO) of Rh­(δ*)/DPhF­(π*) to the lowest unoccupied molecular orbital (LUMO) of bncn­(π*).^16,17^ After rapid intersystem crossing from singlet to triplet excited states, emission spectra at 77 K in CH_3_CN indicate the presence of low-lying triplet ML-LCT (^3^ML-LCT) and metal-centered (MC) states. These states are at sufficiently high energy to prevent fast deactivation, due to a short Rh–Rh bond length (2.4049 Å) that raises the energy of the Rh_2_(σ*) molecular orbital.^16–18^ This low-lying triplet state plays a crucial role in H_2_ production, as catalysis proceeds through successive excited-state redox steps through such triplet state, with electrons being promoted to each bncn­(π*) LUMO at approximately −0.4 to −0.6 V vs Ag/AgCl in CH_3_CN.^16–18^ Recently, Huang et al. also explored incorporating carboxylic acid groups (−COOH) on the formamidine ligands (p-diCOOH-Form = *N*,*N*′-bis­(*p*-carboxyphenyl)-formamidinate) to anchor the DiRh­(II,II) complex to the commonly used p-type semiconductor nickel oxide (NiO).^24^ Nanosecond transient absorption (nsTA) and electrochemical experiments demonstrated that NiO-anchored complexes effectively harvest red light and generate the ^3^ML-LCT excited state, which can inject holes into the valence band (VB) of p-type NiO with a driving force of −0.96 V. This process, coupled with the subsequent localization of electrons on the bncn ligand, allows the DiRh complexes to function both as NiO sensitizers and as HER catalysts. Notably, the DiRh-NiO system outperforms widely used triphenylamine (P1) and other dyes in terms of HER Faradaic efficiency,^25^ highlighting the potential of such DSPEC single-molecule design.^24^ Despite these advancements, several challenges remain, particularly in understanding and optimizing the DiRh/NiO interfacial properties that are key to the stability and efficiency of such a hybrid photocathode.

In this context, we use advanced first-principles calculations, based on Density Functional Theory (DFT),^26^ to explore the structural and electronic characteristics of DiRh/NiO systems, and to analyze the structure–property–function relationships of these interfaces. The strengths and orientations of dye-electrode chemical bonds and their effects on the charge transport at these interfaces are key features in the development of advanced DSPEC designs. Experimental techniques like time-resolved photoluminescence (TRPL) and transient absorption spectroscopy (TAS) show that charge extraction time scales range from subfemtoseconds to the nanoseconds.^24,27–30^ Due to the complexity of these processes, theoretical calculations are essential for dissecting the main electronic features that are crucial in charge dynamics. To this end, we also investigate the heterogeneous DiRh/NiO interface using the projection-operator diabatization (POD) approach, which identifies preferred charge transfer pathways and corresponding time scales.^31–35^ Our findings highlight the presence of strong interactions between the DiRh complexes and p-type NiO, forming stable mono- and bianchored configurations, with the monoanchored complex showing the most promising electronic and charge dynamics features. Our results offer new insights into adsorption characteristics, energy band alignment, and charge dynamics at the DiRh/NiO interface, and highlight the advantages of designing directions toward advanced single-molecule photocatalysts, such as those with asymmetric ligands, for enhanced photoelectrochemical performance.^19–22^

## Methods and Computational Details

Geometry optimizations of molecular *cis*-[Rh_2_(p-diCOOH-Form)_2_(bncn)_2_]^2+^ (labeled as DiRh all through the work) were performed via Density Functional Theory (DFT) at the PBE0 level of theory,^36,37^ including Grimme’s dispersion correction D3 with the Becke–Johnson damping function^38,39^ (D3-BJ), as implemented in Gaussian 16.^40^ The TZVP basis set was used for C, N, H, and O atoms, and the SDD effective core potential (ECP) and basis set were used for Rh. Solvent (acetonitrile, ACN) was considered within the polarizable continuum model (PCM).^41–43^

Geometry optimizations of DiRh­(II,II)/NiO interfaces were performed by spin-polarized DFT calculations with periodic boundary conditions (PBC) employing the light-tier1 basis set of numerical atom-centered orbitals (NAO) for each atom,^44^ as implemented in the Fritz Haber Institute ab initio molecular simulations (FHI-aims) code.^45^ As the self-consistency threshold for electron density convergence, we employed a total energy criterion of 1 × 10^–6^ eV. We employed the PBE^36^ exchange-correlation functional for all geometry optimizations including the Tkatchenko–Scheffler (TS) correction^46,47^ accounting for van der Waals dispersion forces as implemented in the FHI-aims code.^45^ We refine the energetic and electronic analysis at PBE0-D3­(BJ) level of theory^36–39^ considering also the solvent effect by the self-consistent continuum solvation (SCCS) model, as implemented in the CP2K software.^48,49^ Double-ζ basis functions with one set of polarization functions (double-ζ valence polarization (DZVP)) were used as basis sets with a plane wave cutoff of 300 Ry. The Goedecker–Teter–Hutter (GTH) pseudopotentials were used to treat core electrons, while the considered valence electrons were Ni: 3s^2^3p^6^3d^8^4s^2^, O:2s^2^2p^4^, Rh: 4d^8^5s^1^, C: 2s^2^2p^2^, N:2s^2^2p^3^, H:1s^1^. As the self-consistency threshold for electron density convergence, we employed a total energy criterion of 1 × 10^–6^ eV. Since we are dealing with asymmetric systems, a dipole correction is taken into account as implemented in CP2K.^50^ Due to the large system size, all calculations were performed at the gamma point. As a model of the DiRh/NiO interface, we have considered the doubly deprotonated DiRh complex (i.e., featuring two carboxylate −COO^–^ anchoring groups), which not only ensures the lack of net charge of the system under study, but it is also reported to be one of the preferred, effective anchoring modes for NiO.^51–54^

We investigated the charge dynamics at the DiRh/NiO interface by estimating the electron injection time using a simple donor–acceptor model, based on the projection-operator diabatization (POD) approach.^31–35^ Such a method consists of partitioning the Kohn–Sham (or Fock) matrix of the interacting system, expressed in an orthonormal basis (H̅) , into a donor (*D*) and an acceptor (*A*) part, and separately diagonalizing of this matrix blocks, as following:

1 $$\mathrm{H̅}=\left(\begin{array}{ll} \begin{array}{lll} \varepsilon_{D,1} & \cdots & 0\\ \vdots & \ddots & \vdots \\ 0 & \cdots & \varepsilon_{D,N} \end{array} & {\mathrm{H̅}}_{DA}\\ {\mathrm{H̅}}_{AD} & \begin{array}{lll} \varepsilon_{A,1} & \cdots & 0\\ \vdots & \ddots & \vdots \\ 0 & \cdots & \varepsilon_{A,N} \end{array} \end{array}\right)$$

where *ε*_*D*_ and *ε_A_* are the one-electron energies of donor and acceptor localized diabatic states, respectively, while the off-diagonal blocks ( ${\mathrm{H̅}}_{DA}$ , ${\mathrm{H̅}}_{AD}$ ) denote the relative donor–acceptor couplings. In particular for the DiRh/NiO interfaces, we consider the electron transfer from the NiO VB (donor) to DiRh occupied molecular orbitals (MOs) (acceptor), which corresponds to the real process of hole transfer from the DiRh MOs to the NiO VB. The donor–acceptor couplings were computed at the PBE0-D3­(BJ) level of theory,^31–34^ as implemented by Futera and Blumberger^32^ in the CP2K software.^49^

## Results and Discussion

### Molecular Properties of the *cis*-[Rh_2_(p-diCOOH-Form)_2_(bncn)_2_]^2**+**^(DiRh) Complex

First, we explore the molecular properties of the *cis*-[Rh_2_(p-diCOOH-Form)_2_(bncn)_2_]^2+^ (DiRh) complex in an acetonitrile (ACN) solution. Since previous theoretical investigations highlighted the possible presence of solvent molecule at axial positions,^15^ we include two ACN molecules coordinated to the Rh atoms. Figure 1a shows the computed PBE0-D3­(BJ) minimum energy structures of the DiRh complex in its singlet ground state [DiRh]^S^ and in the lowest triplet excited state [DiRh]^T^, which play a crucial role in hydrogen production.

**Figure 1:**
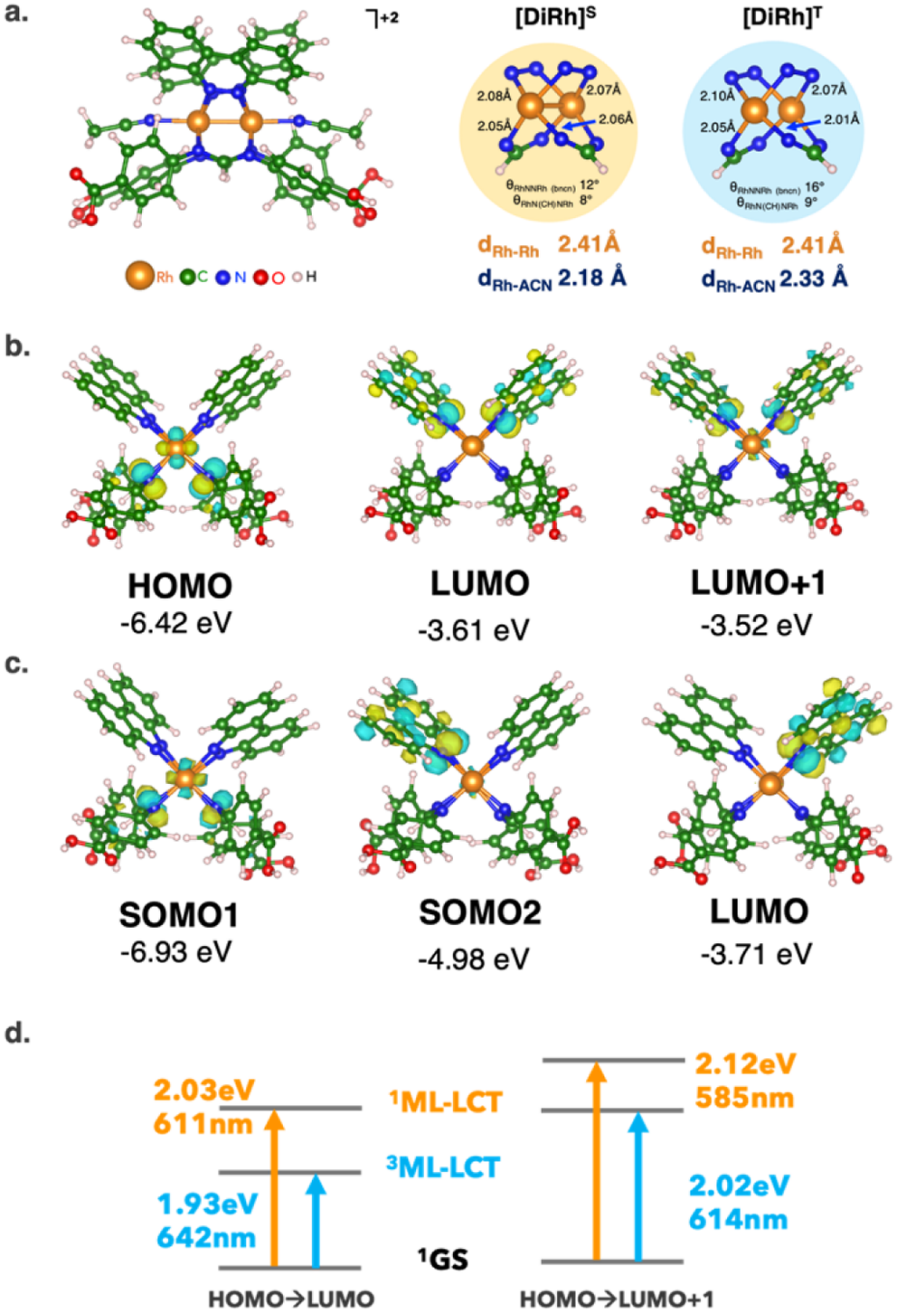
**(a)** Minimum energy structure at the PBE0-D3­(BJ) level of theory of the DiRh complex in the singlet ground state ([DiRh]^S^) and the lowest triplet excited state ([DiRh]^T^). **(b)** DiRh molecular orbitals (MOs) of singlet ground (**orange panel**) and triplet excited states (**blue panel**). Isodensity surfaces are depicted in yellow and cyan for positive and negative values, respectively. Isosurface value: 0.03 au. **(c)** Computed DiRh maximum absorption wavelength (*λ*_max_) in acetonitrile at the PBE0-D3­(BJ) level of theory via TD-DFT calculations. **(d)** Singlet and triplet metal/ligand-to-ligand charge transfer (^1^ML-LCT: orange, ^3^ML-LCT: light blue) involving the HOMO/LUMO and HOMO/LUMO + 1 charge transfer are shown. Atomic color code: Rh (orange), C (green), N (blue), O (red), H (white).

The structural features of [DiRh]^S^ and [DiRh]^T^ are very similar, with Rh–Rh bond lengths of ∼2.41 Å, which are very close to the experimental value of 2.4049 Å.^16^ Only a slight elongation of ∼0.2 Å is found at the Rh–ACN axial bond in [DiRh]^T^, suggesting a more liable solvent complex bonding in the excited state, potentially leading to the creation of an axial free site for the formation of a Rh–H hydride intermediate during HER photocatalysis.^24^

Electronic analysis of DiRh molecular orbitals (MOs) shows that the HOMO and LUMO align well with those of similar complexes.^55,56^ In [DiRh]^S^, the HOMO is predominantly localized on Rh­(δ*)/p-diCOOH-Form­(π*), while the LUMO is localized on bncn (π*), another bncn (π*) MO (LUMO + 1) is found at only ∼0.1 eV above the LUMO. These MOs are also involved in [DiRh]^T^ frontier orbitals: the unpaired electrons are found in two SOMOs (singly occupied molecular orbitals), localized one on Rh (δ*)/p-diCOOH-Form­(π*) and the other on bncn (π*) (see Figure 1b,c).

Regarding optical transition, we compute the maximum absorption wavelength (*λ*_max_) of the DiRh complex via Time-Dependent DFT (TD-DFT) at the PBE0 level of theory (Figure 1d). At this level of theory, we find an intense high-energy absorption band at 611 nm attributed to singlet metal-to-ligand charge transfer (^1^ML-LCT). This MLCT involves electron transfer from the Rh­(δ*)/p-diCOOH-Form­(π*) HOMO to the bncn­(π*) LUMO and the predicted value is consistent with the experimental absorption spectrum in ACN (*λ*_exp_ = 605 nm). To assess the reliability of the chosen functional, we compared the predicted absorption wavelengths at different DFT levels of theory (Table 1). While standard GGA or hybrid functionals often fail to accurately reproduce excitation energies due to their sensitivity to long-range interactions in TD-DFT, we found that the PBE0 functional, combined with a static solvation model, provided the best agreement with experimental data. This accuracy can be rationalized by the nature of the excitation, which is dominated by a HOMO–LUMO transition known to be less sensitive to long-range effects.^57^ Moreover, it is already known that PBE0 benefits from favorable error cancellation in describing ^1^MLCT states.^58^ Overall, our findings support the use of PBE0 with implicit solvation as a computationally efficient and accurate method for investigating the optical and electronic properties of DiRh complexes.

**Table 1:** Computed DiRh Maximum Absorption Wavelength (λ_max_ in eV and nm) with Oscillator Strength (*f*) in Acetonitrile for Singlet MLCT Involving the HOMO/LUMO at the TD-DFT Level of Theory with Different Functionals

DFT Functional	λ_max_ (eV)	λ_max_ (nm)	*f*
**PBE**	1.34	925.00	0.09
**B3LYP**	1.88	659.27	0.06
**PBE0**	2.03	611.31	0.06
**CAM-B3LYP**	2.71	457.09	0.07
**wB97XD**	2.75	450.47	0.08
**M06-2X**	2.82	439.62	0.01
**BHandHLYP**	2.96	418.87	0.01
**LC-wHPBE**	3.31	374.44	0.11

Via such an approach, an analogous ML-LCT for [DiRh­(ACN)_2_]^T^ (^3^ML-LCT) is calculated at a lower energy with *λ*_max_ = 642 nm. These results agree with the emission spectra at 77 K in ACN, showing the presence of a low-lying ^3^ML-LCT state that is an excited state of significant importance in hydrogen production.^24^ Electrochemical experiments show that HER catalysis proceeds through successive excited-state redox steps, with each step involving the addition of an electron to the bncn­(π*) LUMO, as

2 $${[DiRh]}^{+2}\overset{E_{red}\left(1\right)}{\to }{[DiRh]}^{+1}\overset{E_{red}\left(2\right)}{\to }{[DiRh]}^{0}$$

We computed the reduction potentials (*E*_red_) of the DiRh complex from both singlet ground and triplet excited state, given as

3 $$E_{red}=-\frac{G_{red}}{nF}-4.43\;V-0.22\;V$$

where *G*_red_ is the difference in free energies (*G*_red_ = *G^n^* – *G^n^* ^–1^) between successive reduced states of the DiRh molecule (*G* ^+2^, *G* ^+1^, and *G*°, respectively), *n* is the number of electrons transferred (i.e., *n* = 1), *F* is the Faraday constant (1 eV/V), while −4.43 and −0.22 V are the voltage shifts relative to the vacuum with the normal hydrogen electrode (NHE) and the Ag/AgCl electrode vs NHE, respectively.^59^

We find that *E*_red_(1) and *E*_red_(2) are approximately −0.35 and −1.22 V (vs Ag/AgCl) for the singlet ground state and −1.0 and −0.60 V (vs Ag/AgCl) for the triplet excited state, respectively. Comparing our results with the electrochemical experimental *E*_red_(1) and *E*_red_(2) values for the complex under investigation (−0.38/–0.59 V vs Ag/AgCl in acetonitrile)^24^ and analogous DiRh complexes,^16–18^ we conclude that the first reduction likely occurs from the singlet ground state, while the second reduction from the stable reduced triplet state.

Concerning the ability of accepting the electron from the NiO substrate, the inner reorganization energy (λ) in vacuo provides a qualitative descriptor for the charge transfer process.^11,60–62^ This quantity represents the energy required to reorganize the geometry of the molecule after gaining one electron from the NiO surface. We determined λ via the adiabatic potential energy surface method^63^ as follows:

4 $$\lambda =\left(E_{-}^{*}-E_{-}\right)$$

where $E_{-}^{*}$ represents the total energy of the reduced molecule $\left({[DiRh]}^{+1/0}\right)$ at the optimized geometry of molecule ${[DiRh]}^{+2/+1}$ , and $E_{-}$ is the total energy of the reduced molecule $\left({[DiRh]}^{+1/0}\right)$ . The low λ (∼0.1–0.2 eV, see Figure S1) suggests a slight energy barrier for the photocatalyst regeneration by the p-type substrate. Overall, such results, in agreement with experimental findings,^24^ validate the promising features of the DiRh complex as a compelling candidate for dye-catalyst applications in DSPEC devices, particularly for charge transport.

### DiRh/NiO Interface: Method Validation

The molecular properties of DiRh are thus addressed considering its adsorption onto the defective NiO surface (NiO:V_Ni_) to model the p-type nature of NiO-based electrodes. We consider two different possible isoelectronic configurations: (1) s-DiRh, where two carboxylic acid groups on the same p-diCOOH-Form ligand moiety are deprotonated (*cis*-[Rh_2_(p-diCOO^–^-Form)­(p-diCOOH-Form)­(bncn)_2_], s-DiRh in Figure 2a); (2) a-DiRh, where the two deprotonated carboxylic acid groups are those of two different p-diCOOH-Form ligands, in alternate positions (a-DiRh: *cis*-[Rh_2_(p-COOHCOO^–^-Form)_2_(bncn)_2_], a-DiRh in Figure 2a). These s-/a-DiRh complexes exhibit minimal structural discrepancies with the fully protonated *cis*-[Rh_2_(p-diCOOH-Form)_2_(bncn)_2_]^2+^ complex, but their electronic features are slightly different. While the HOMO and LUMO for all species are consistently localized on Rh (δ*)/p-diCOOH-Form­(π*) and bncn­(π*), respectively (see Figure 2b,c), the formation of −COO^–^ reduces the HOMO–LUMO gap, which is approximately 1.4 eV in both deprotonated s/a-DiRh complexes, as previously suggested.^24^

**Figure 2:**
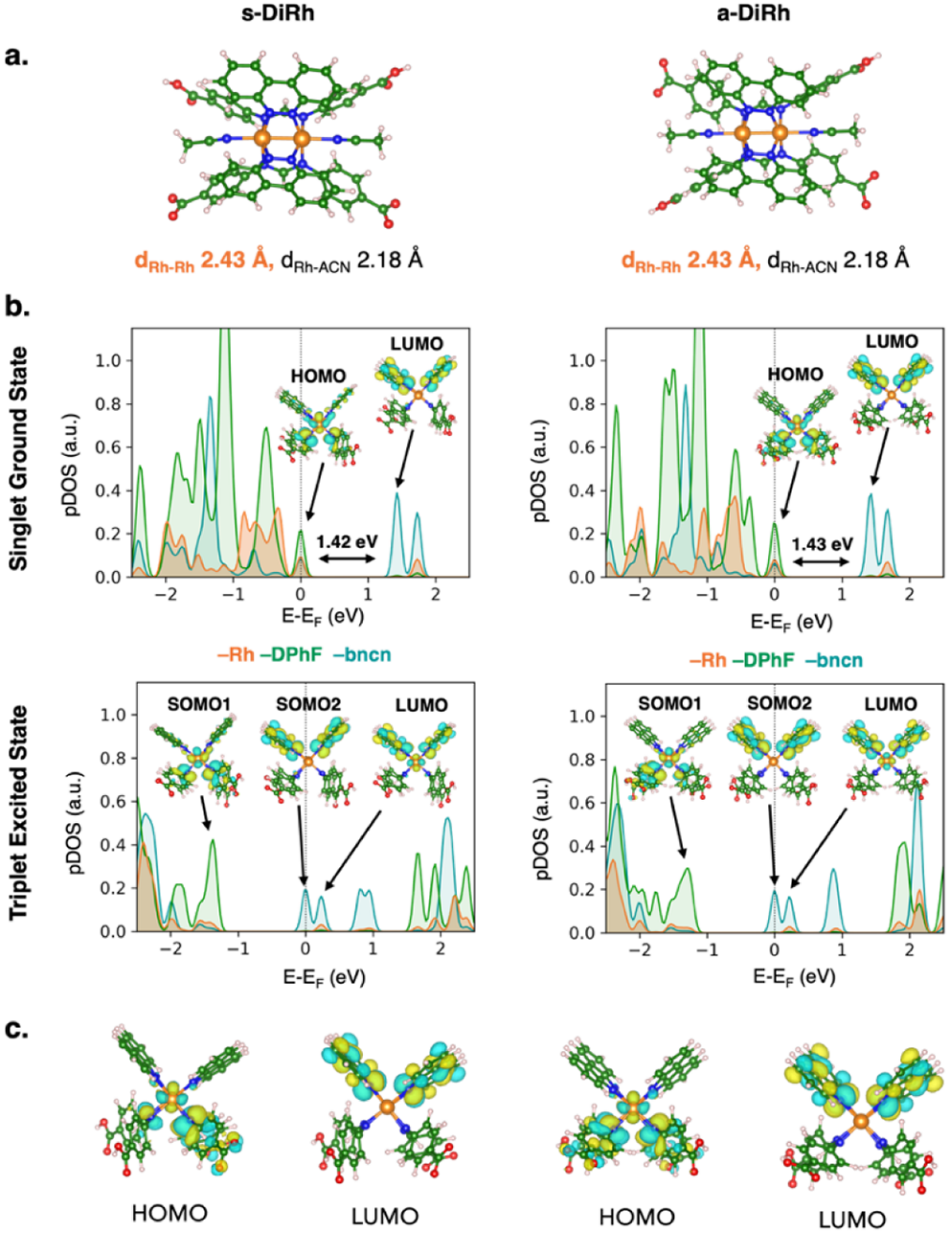
**(a)** Minimum energy structure at the PBE0-D3­(BJ) level of theory of the DiRh complex with the PBC framework (in a box of 25 Å × 25 Å × 40 Å): **(left panel)** s-DiRh, where two carboxylic acid groups on the same ligand moiety are both deprotonated; **(right panel)** a-DiRh, where the two deprotonated carboxylic acid groups are those of two different ligands, in alternate positions. **(b)** Projected density of states (pDOS) at the PBE0-D3­(BJ) level of theory of s-DiRh (**left panel**) and a-DiRh (**right panel**) in the presence of acetonitrile as a solvent. Both pDOS of singlet ground and triplet excited states with respective HOMO, LUMO, and SOMO are depicted. **(c)** HOMO and LUMO computed at the PBE0-D3­(BJ) level of theory in acetonitrile as a solvent via Gaussian software. Isodensity surfaces are depicted as yellow and cyan for positive and negative values, respectively. Isosurface value: 0.03 au. Atomic color code: Rh (orange), C (green), N (blue), O (red), H (white). pDOS color code: Rh (orange), bncn ligands (teal), DPhF (p-diCOOH-Form), ligands (dark green).

Accurate modeling of the NiO substrate is equally important, we first assessed the reliability of the chosen functional for DiRh complexes, the PBE0, to predict the antiferromagnetic ground state of NiO.^64^ Standard GGA functionals are well known to severely underestimate the NiO band gap, whereas the GGA + U approach improves the localization of Ni 3d states but suffers from dependence on empirically chosen *U* values, which can limit its application.^51,65–69^ In contrast, hybrid functionals such as PBE0 have been shown to accurately reproduce both the experimental band gap and magnetic ordering of NiO.^64,70,71^ Figure 3a shows the projected density of states (pDOS), spin density, and spin magnetic moment on Ni atoms from Mulliken Population Analysis (μ_Ni_ + 1.67/–1.67) for the optimized 2 × 2 × 2 NiO bulk supercell with lattice constant 16.75 Å (4.18 Å/fu) in each direction. This electronic analysis confirms that PBE0 correctly predicts the antiferromagnetic behavior of NiO, and the computed band gap is in excellent agreement with previous reports.^72,73^

**Figure 3:**
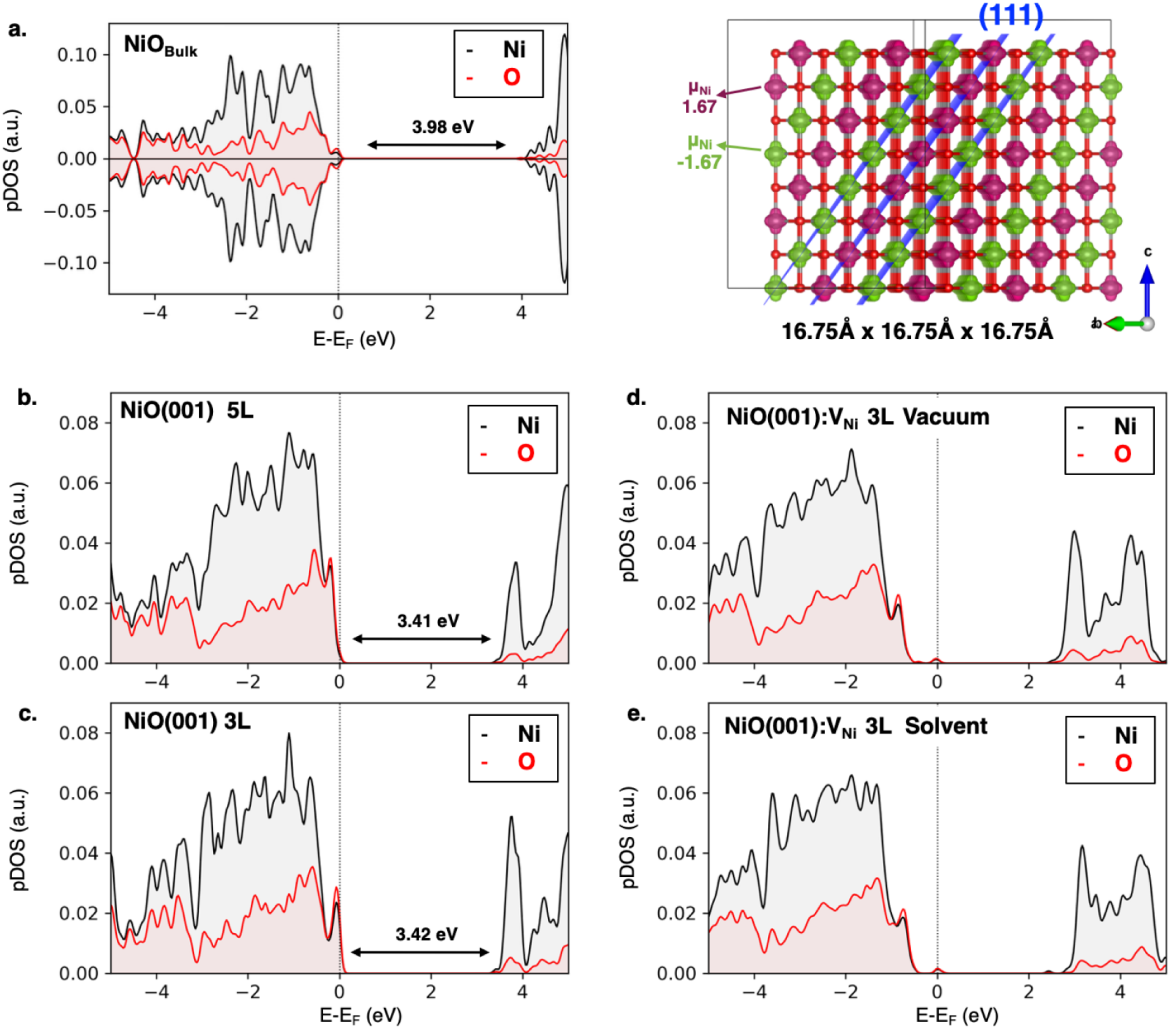
**(a)** Projected density of states (pDOS) **(left panel)**, spin magnetic moment on Ni atoms from Mulliken Population Analysis (μ_Ni_) and spin density **(right panel)** at the PBE0-D3­(BJ) level of theory of the optimized NiO bulk structure. Magenta and green regions denote alpha and beta spin density, respectively. Isosurface value: 0.03 au. pDOS at the PBE0-D3­(BJ) level of theory of pristine (001)-NiO surfaces: **(b)** 5 layers (5L) slab and **(c)** 3 layers (3L) slab in vacuum, and of 3L defective (001)-NiO (NiO:V_Ni_) in **(d)** vacuum and **(e)** acetonitrile solvent.

To investigate the DiRh/NiO interfaces, we employed a three-layer 3 × 3 supercell slab model of the most exposed NiO (001) surface with 35 Å of vacuum along the c direction. Such a model prevents interactions between periodic images of DiRh complexes. Plus, this supercell size ensures an accurate representation of the electronic structure of the pristine NiO(001) surface, with the intrinsic band gap of 3.4 eV. This was confirmed by comparing the pDOS calculated at the PBE0-D3­(BJ) level of theory for three- and five-layer slabs (3L vs 5L, Figure 3b–e). All geometry optimizations are performed while keeping the third layer frozen. Figure 3 also illustrates the pDOS of defective surfaces with a Ni vacancy, both in a vacuum and in the presence of acetonitrile as a solvent. The introduction of the defect generates an intragap state, predominantly localized on oxygen atoms.^74^ Overall, the combined analysis of the two DiRh configurations and the electronic properties of NiO bulk, pristine, and defective NiO(001) surfaces validates the structural models employed and supports the use of the PBE0 functional for accurately describing the interfacial electronic structure.

### DiRh/NiO Interface: Structural, Electronic, and Charge Transfer Features

Within the considered s-DiRh and a-DiRh complexes, we can explore two distinct anchoring modes at NiO interfaces: monoanchored via the single deprotonated p-diCOO^–^-Form ligand for the s-DiRh and the bianchored via both p-COOHCOO^–^-Form ligands for the a-DiRh (Figure 4a).

**Figure 4:**
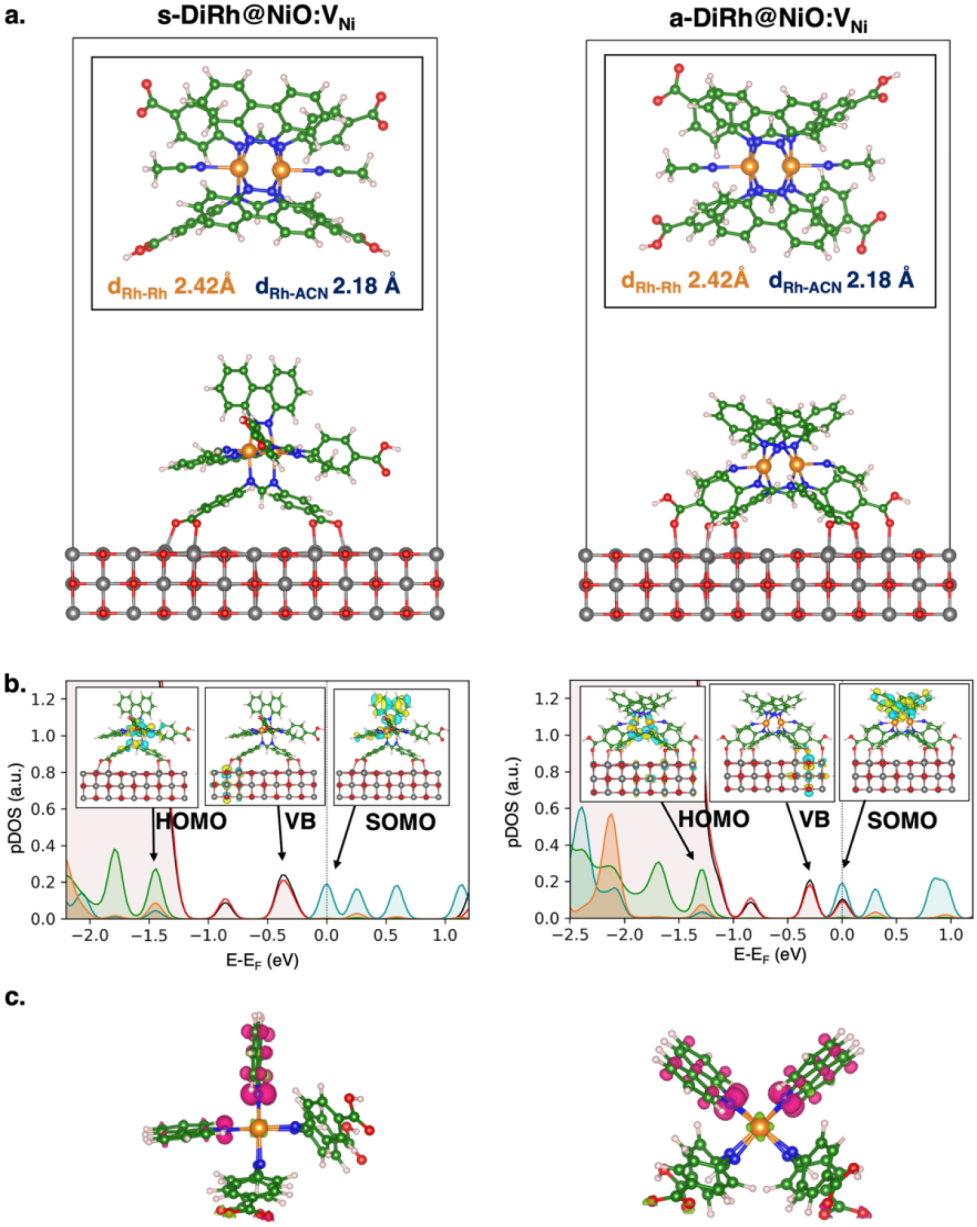
**(a)** Minimum energy structures of DiRh/NiO interfaces in the singlet state with the computed adsorption energies (*E*_ads_) at the PBE0-D3­(BJ) level of theory. Monoanchored s-DiRh/NiO (**left panel**) and bianchored a-DiRh/NiO (**right panel**) configurations. Specifically, s-DiRh has two carboxylic acid groups on the same ligand moiety deprotonated (*cis*-[Rh_2_(p-diCOO^–^-Form)­(p-diCOOH-Form)­(bncn)_2_]), while in a-DiRh the two deprotonated carboxylic acid groups are those of two different ligands, in alternate positions (*cis*-[Rh_2_(p-COOHCOO^–^-Form)_2_(bncn)_2_]). The Rh–Rh and Rh–ACN bond lengths of complexes at the NiO interface are reported in the figure. **(b)** Projected density of states (pDOS) at the PBE0-D3­(BJ) level of theory of triplet excited states of s-DiRh (**left panel**) and a-DiRh (**right panel**) in the presence of acetonitrile as a solvent. The high-occupied DiRh MO (HOMO), NiO valence state (VB), and single-occupied DiRh MO (SOMO) are depicted. Isodensity surfaces are depicted as yellow and cyan for positive and negative values, respectively (isosurface value: 0.03 au). **(c)** Spin density of the triplet excited state of: s-DiRh/NiO:V_Ni_ **(left panel)** and a-DiRh/s-DiRh/NiO:V_Ni_ **(right panel)** in acetonitrile solvent. Magenta and green regions denote alpha and beta spin density, respectively (isosurface value: 0.005 au). Atomic color code: Ni (gray), O (red), Rh (orange), C (green), N (blue), H (white). pDOS color code: Ni (black), NiO surface O (red), Rh (orange), bncn ligands (teal), and DPhF (p-diCOOH-Form) ligands (dark green).

For each configuration, we computed adsorption energies (*E*_ads_) at the PBE0-D3­(BJ) level of theory as

5 $$E_{ads}=E_{DiRh/NiO}-E_{NiO}-E_{DiRh}$$

where $E_{DiRh/NiO}$ , *E*_NiO_, and *E*_DiRh_ are the energy of the bound systems, the bare NiO surfaces and the isolated s/a-DiRh neutral complexes. The a-DiRh/NiO:V_Ni_ interface in acetonitrile solution is slightly more stable than the s-DiRh/NiO by ∼0.5 eV, with *E*_ads_ being −5.86 and −5.38 eV for a-DiRh and s-DiRh, respectively. The origin of this difference is in the different complex-surface binding (Figure 4a), with the monoanchored s-DiRh attached with two strong bidentate −COO^–^···Ni bonds, with an equilibrium binding distance between the carboxylic oxygen and nickel of 2.05 Å. The bianchored a-DiRh, instead, is attached to the NiO surface with the two −COOH in a monodentate binding mode and two −COO^–^ groups in a bidentate binding mode with equilibrium bond distances with surface Ni of 2.20 and 2.05 Å for the −COOH and −COO^–^ groups, respectively. The investigation considering the DiRh complex in its excited triplet state provides similar results, with *E*_ads_ of −5.36 and −5.06 eV for a-DiRh and s-DiRh, respectively. Electronic and structural analysis of the DiRh/NiO interfaces (see Figure S2) shows a slight charge redistribution localized on the Ni–O_DiRh_ bonds and no significant structural distortions of the NiO surface upon interface formation. Overall, these findings suggest that the strong interaction between the DiRh complexes and the NiO surface is primarily a result of the electronic interactions between the −COO^–^ groups and the surface Ni atoms.

We particularly focus on the triplet excited state, which plays a primary role in charge transfer processes at DiRh/NiO interfaces and in hydrogen production.^21^ Analysis on the high-resolved pDOS and frontier molecular orbitals of the entire systems (computed as band-decomposed charge density of the highest occupied/lowest unoccupied bands of adsorbed complexes on NiO, Figure 4b) of s/a-DiRh/NiO:V_Ni_ interfaces in the triplet state exhibits a singly occupied molecular orbital on the bncn (π*) MO. Such results are also confirmed by the analysis of the electronic spin densities in Figure 4c and suggest a faster electron reorganization at DiRh/NiO interfaces. Notably, the bncn (π*) MO in s-DiRh­(ACN)_2_ is conveniently located on the bncn ligand that is further away from the electrode surface, thus minimizing undesired charge recombination (Figure 4b).

In all cases, the high-resolution pDOS and frontier MOs (Figures 4b) highlight a proper alignment between the DiRh frontier orbitals and the NiO valence band that is suitable for effective hole injection from the catalysts to the p-type electrode. There are valence states primarily given by defective NiO surface states close to the COO–Ni bonds above the s/a-DiRh Rh (δ*)/p-diCOOH-Form­(π*) MO, which is the HOMO of the isolated complexes (HOMO, Figure 4b). Additionally, the s-DiRh/NiO interfaces exhibit higher driving forces (0.60 eV) compared to those of the a-DiRh/NiO interfaces (0.45 eV), respectively (Figure 5a). Similar results are found for the singlet state (Figure S3).

**Figure 5:**
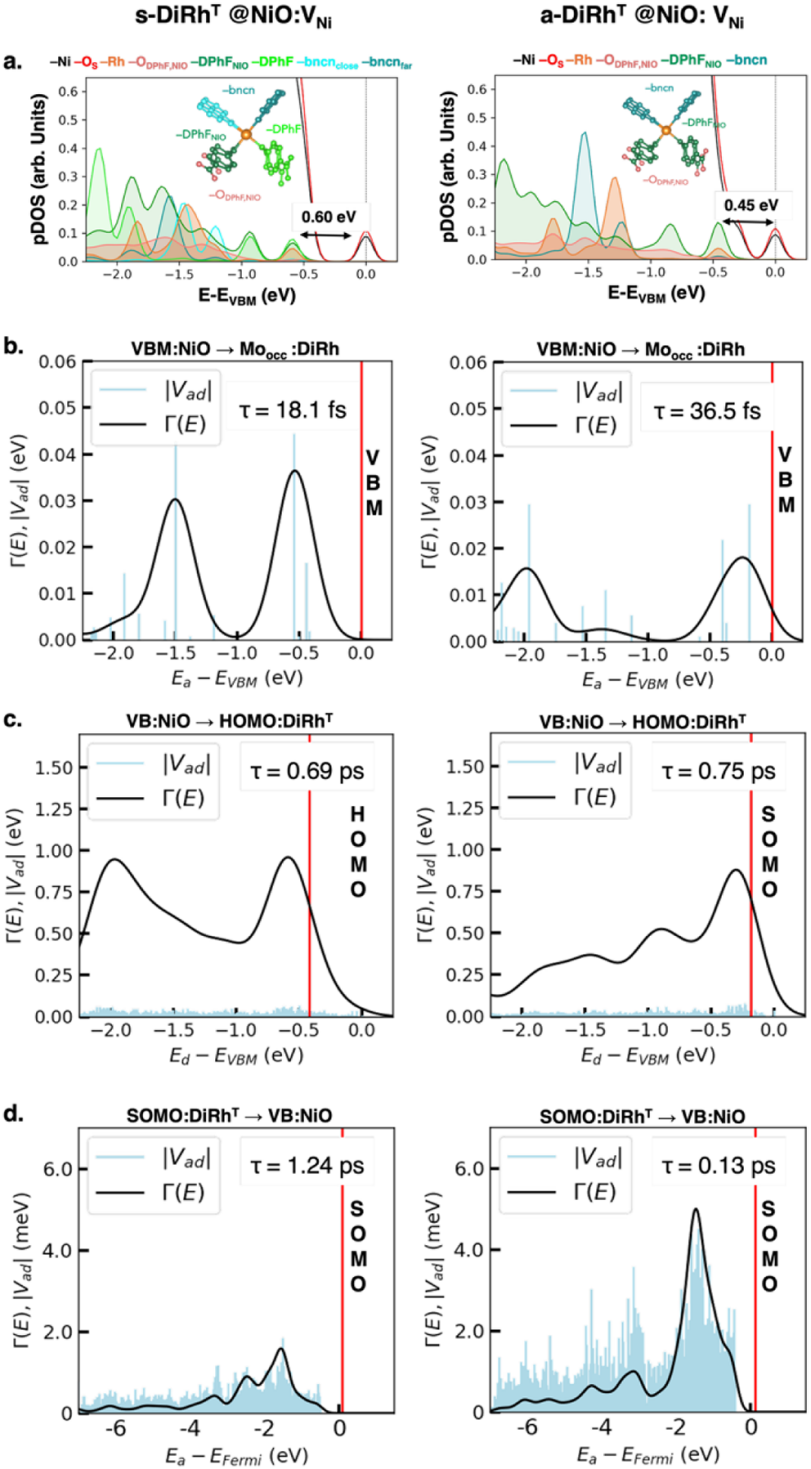
**(a)** High-resolution projected density of states (pDOS) of s-DiRh **(left panel)** and a-DiRh **(right panel)** complexes at the NiO:V_Ni_ surface in the triplet excited state. **(b,c)** Coupling elements, spectral functions, and hole injection time evaluated at the PBE0-D3­(BJ) level of theory for the valence band maximum (VBM) of NiO with all the occupied states of s-DiRh and a-DiRh complexes in the triplet state. **(c)** Coupling elements, spectral functions, and hole injection time evaluated at the PBE0-D3­(BJ) level of theory for the highest-occupied molecular orbital (HOMO) of s-DiRh and a-DiRh complexes in the triplet state to all valence band state of NiO. **(d)** Coupling elements, spectral functions and electron injection time evaluated at the PBE0-D3 (BJ) level of theory for the SOMO (see Figure 4b) of DiRh with all the valence states of NiO in the triplet state. Coupling elements and spectral functions are represented as a function of the acceptor state energy, while donor state energy is indicated by a red vertical line. pDOS color code: Ni (black), O_S_ (red), Rh (orange), bncn ligands (teal), DPhF (p-diCOOH-Form) ligands (dark green).

Motivated by these findings, we explored the charge dynamics at DiRh/NiO:V_Ni_ interfaces by computing hole injection rates and identifying the preferential channels for charge transfer via the POD approach.^31–34^ Since the hole transfer process from the DiRh MOs to the NiO VB is conceptually equivalent to an electron transfer in the opposite direction, we investigate the electron injection from the NiO VB (donor) to DiRh occupied MOs (acceptor). From donor–acceptor coupling matrix elements (*V*_ad_), we calculated the spectral function (Γ_*d*_(*E*)) defined as

6 $$\Gamma_{d}\left(E\right)=2\pi \underset{a}{\sum }{|V_{ad}|}^{2}\delta \left(E-\varepsilon_{a}\right)$$

where *V*_ad_ is the electronic coupling matrix element between the diabatic donor *d* state of NiO VB and the acceptor *a* state of s/a-DiRh occupied MOs, with energy *ε_a_*. This function can be regarded as an estimate of the donor state decay width and provides information about the charge transfer process time scale. The time for hole injection can be calculated in terms of the spectral function as τ = h̅/Γ.^31–34^ The POD method applied to ground-state DFT-optimized structures is already established as a promising approach to estimate the injection times and in particularly for material comparisons for large periodic systems.^30,31^ Also, we must note that dynamic effects and thermal fluctuations, which could affect the localization of electronic states and coupling matrix elements, are neglected here in this work but represent an important feature to address in future studies.

Figure 5b shows the coupling elements and spectral functions for electron injection from the valence band maximum (VBM) of NiO into all of the occupied states of s/a-DiRh complexes in the triplet states. In agreement with experimental TRPL data for other dyes at NiO interfaces,^27–29^ computed hole injection times are on the femtosecond scale. However, when comparing the two distinct s/a-DiRh/NiO interfaces, we found that the s-DiRh complex exhibits a faster hole injection than that of a-DiRh (∼18 fs vs ∼36 fs, respectively). Similar results are found for the singlet state (see Figure S4).

The charge transfer process involves the HOMO of the DiRh complex and inner states of the DiRh complex, with energy levels ranging from −2 to −1 eV. Analysis of pDOS denotes that the occupied DiRh states in this energy range are localized on Rh atoms, suggesting that the Rh MOs play a crucial role in the charge transfer process at NiO interfaces. This suggests that the Rh atoms, which receive the electron together with the DiRh moiety, are involved in both photoexcitation and the reduction of the DiRh complex during H_2_ evolution photocatalysis. On the other hand, the coupling between the HOMO of s/a-DiRh and the valence states of defective NiO surfaces suggests ultrafast hole transfer on the subfemtosecond time scale, driven by inner states of the NiO valence band (Figure 5c).

To quantitatively estimate charge recombination, we also evaluated electron injection from the DiRh SOMO (Figure 4b) to the NiO valence states (Figure 5d). In this case, we considered the DiRh occupied MOs as the donor and NiO valence states as the acceptor. Our analysis reveals that such electron injection occurs on the picosecond time scale, with a-DiRh exhibiting a faster injection rate than s-DiRh (0.13 ps vs 1.24 ps, respectively). This trend is consistent with the SOMO localization closer to the electrode surface in the a-DiRh/NiO interfaces (Figure 4b).

Overall, such findings suggest that the monoanchored s-DiRh/NiO interface is the most promising for facilitating faster hole transfer, owing to its stronger coupling with the NiO valence band and its slower charge recombination. These results indicate that a monoanchored configuration can enhance the efficiency of charge separation and reduce potential recombination losses at the interface.

## Conclusions

We have presented a thorough first-principles investigation of the structural and electronic properties of DiRh/NiO interfaces. Molecular orbital analysis of the DiRh complexes confirmed that the HOMO is predominantly located on Rh­(δ*)/p-diCOOH-Form­(π*), while the LUMO is localized on the bncn (π*) ligand, consistent with analogous complexes reported in the literature.^55,56^ TD-DFT calculations and computed reduction potentials confirmed the presence of a low-lying triplet excited state with minimal structural differences compared to the singlet ground state. Noteworthy, our analysis validates PBE0 as a high-accuracy functional for modeling both molecular (DiRh) and extended (NiO) systems, ensuring consistent and reliable treatment of the interface.

With this hybrid DFT level of theory and a supercell slab model for the p-type NiO electrode, we explored multiple DiRh anchoring modes. Strong COO^–^–Ni bonds stabilize both mono- and bianchored configurations, with the monoanchored (s-DiRh) complex exhibiting particularly favorable electronic coupling to the NiO valence band. Effective POD analysis predicts ultrafast hole injection, particularly for the monoanchored s-DiRh complex, supported by favorable alignment of Rh-based orbitals with the NiO valence band. These findings point to asymmetric monoanchored configurations as an effective design strategy to combine ultrafast hole injection with reduced charge recombination.

Overall, our results provide new atomistic insights into the complex interfacial properties of DiRh/NiO systems, underscoring the potential of DiRh complexes for applications in dye-sensitized photocathodes for hydrogen evolution. More broadly, this work validates hybrid-functional DFT combined with POD analysis as a powerful and transferable computational framework for capturing the structural, electronic, and charge transfer properties of complex electrode–molecule interfaces, providing key atomistic insights to guide the design of next-generation photoelectrocatalytic systems.

## Supplementary Material



## Notes

Electronic features of DiRh complex, charge difference plot and structural analysis of DiRh/NiO interfaces. Projected density of states, coupling elements, spectral functions and hole injection time for the singlet ground state of DiRh/NiO interfaces. XYZ coordinates of DiRh/NiO interfaces (PDF)
