# Supplementary material for: Electronic Structure and Interfacial Hole Transfer in a Di-Rhodium Photocatalyst on a p‑Type NiO Electrode
Source: J Phys Chem C Nanomater Interfaces. 2025 Oct 28;129(44):20025–34. doi: 10.1021/acs.jpcc.5c05879 (PMC12599078; doi:10.1021/acs.jpcc.5c05879)
Supplement: Supplementary file 1 [file jp5c05879_si_001.pdf]

Supporting Information

Electronic Structure and Interfacial Hole Transfer in a Di-Rhodium Photocatalyst on a P-Type Nio Electrode.

Francesca Fasulo,[a] Adriana Pecoraro,[a] Ana B. Muñoz-García,[a,*] and Michele Pavone [b,*]

(a) Department of Physics "Ettore Pancini", University of Naples Federico II, Complesso Universitario Monte Sant'Angelo Via Cinita 21, 80126 Naples, Italy

(b) Department of Chemical Sciences, University of Naples Federico II, Complesso Universitario Monte Sant'Angelo Via Cinita 21, 80126 Naples, Italy

* Corresponding authors: ABMG anabelen.munozgarcia@unina.it, MP michele.pavone@unina.it

Electronic features of DiRh complex.

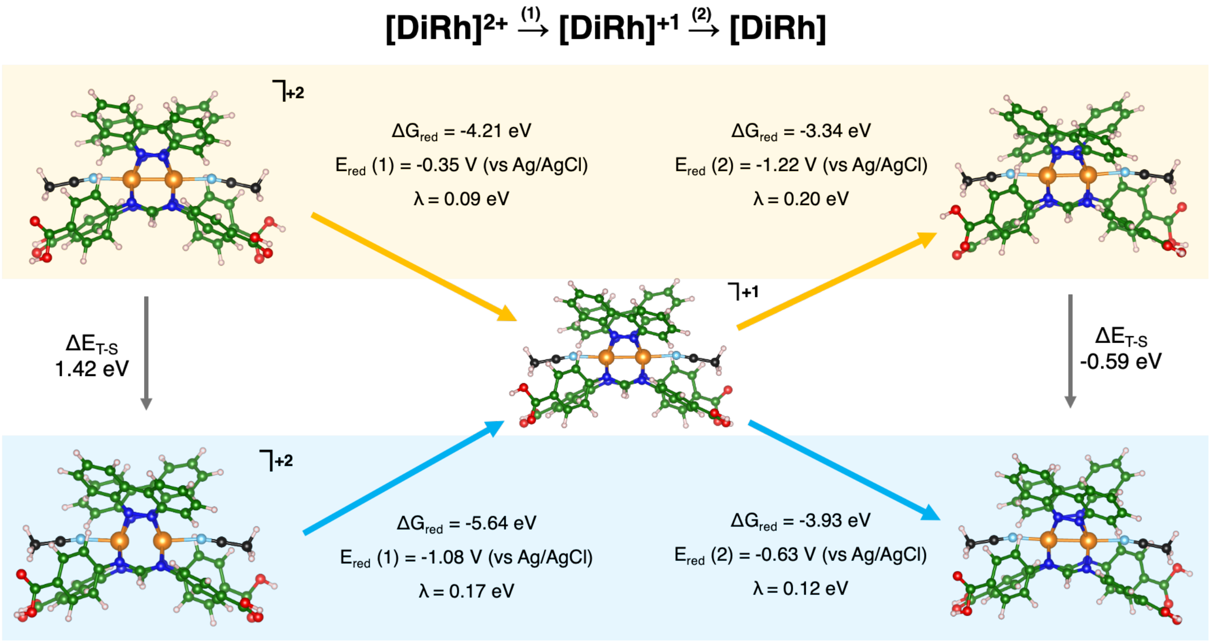

Figure S1. Free energies (G_{red}) and potentials (E_{red}) (eq. 3) at PBE0-D3(BJ) level of theory of successive DiRh complex reduction in both singlet ground and triplet excited states. The inner reorganization energy (λ) (eq. 4) at PBE0-D3(BJ) level of theory are also listed. Atomic color code: Rh (orange), C (green), N (blues), O (red), H (white).

Electronic and structural analysis of DiRh/NiO interfaces.

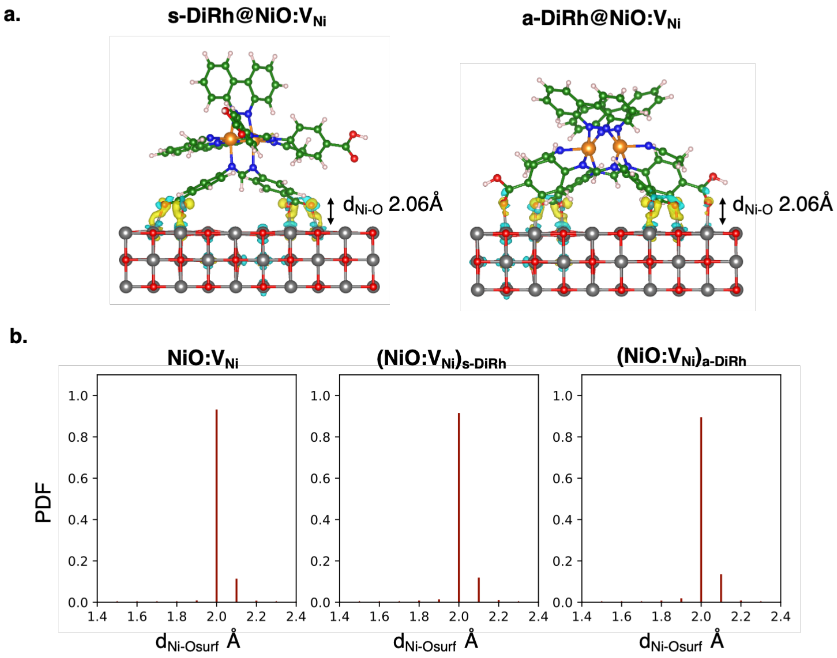

Figure S2. (a) Charge difference plot at PBE0-D3(BJ) level of theory of s-DiRh (**left panel**) and a-DiRh (**right panel**) with singlet spin multiplicity in the presence of acetonitrile as a solvent. The mean value of bond lengths between Ni surface atoms and O atoms of DiRh complexes ($d_{Ni\text{-}O}$) are reported. Isodensity surfaces are depicted as yellow and cyan for positive and negative values, respectively (isosurface values 0.005 a.u.). **(b)** Pair-distribution function (PDF) of the Ni-O surfaces distances ($d_{Ni\text{-}Osurf}$) for the defective NiO surface (NiO:V_{Ni}), NiO surface at s-DiRh and a-DiRh interfaces ($(NiO{:}V_{Ni})_{s/a\text{-}DiRh}$). Atom color code: Ni (grey), O(red), Rh (orange), C (green), N (blues), H (white). PDF color code: $d_{Ni\text{-}Osurf}$ (red).

DiRh/NiO interfaces in the ground singlet state.

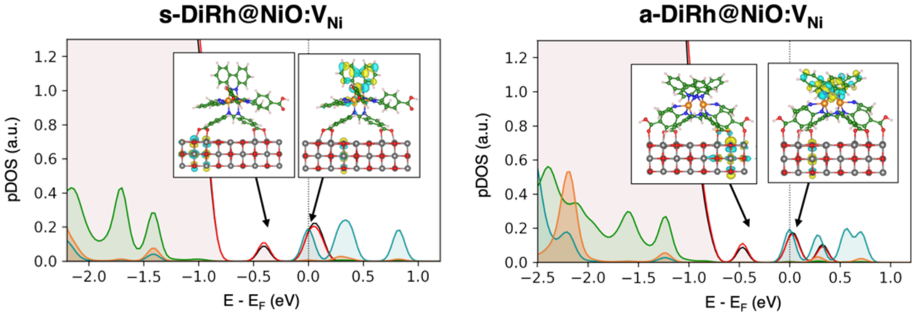

Figure S3. Projected density of states (pDOS) at PBE0-D3(BJ) level of theory of s-DiRh (**left panel**) and a-DiRh (**right panel**) with singlet spin multiplicity in the presence of acetonitrile as a solvent. The NiO valence state (VB) and single-occupied DiRh MO (SOMO) are depicted. Isodensity surfaces are depicted as yellow and cyan for positive and negative values, respectively (isosurface values 0.03 a.u.). Atom color code: Ni (grey), O(red), Rh (orange), C (green), N (blues), H (white). pDOS color code: Ni (grey), NiO surface O(red), Rh(orange), bncn ligands (teal), DPhF (p-diCOOH-Form) ligands (dark green).

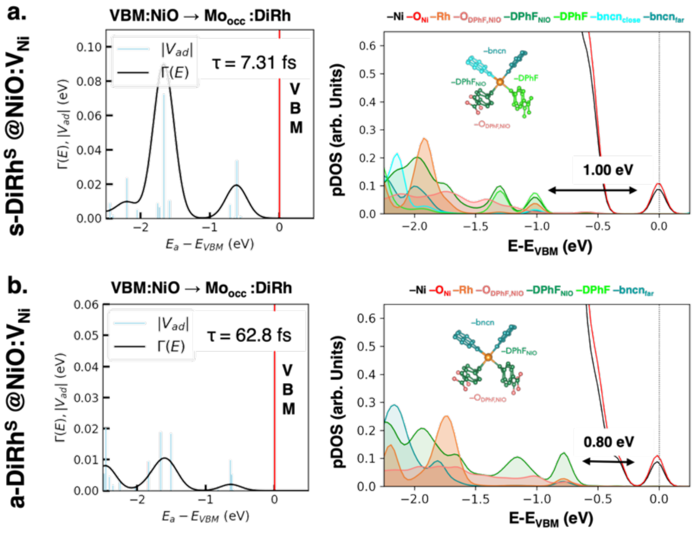

Figure S4. (a-b) Coupling elements, spectral functions and hole injection time evaluated at PBE0-D3(BJ) level of theory for valence band maximum (VBM) of NiO with all the occupied states of s-DiRh **(a)** and a-DiRh **(b)** complexes in the singlet state **(left panel)**. These quantities are represented as a function of the acceptor states energy (DiRh MOs), while donor state energy (NiO VBM) is indicated by a red vertical line. High-resolution projected density of states (pDOS) of s-DiRh **(a)** and a-DiRh **(b)** complexes at NiO:V$_{Ni}$ surface in the singlet state are also showed **(right panel)**. pDOS color code: Ni (black), O$_S$(red), Rh (orange), bncn ligands (teal), DPhF (p-diCOOH-Form) ligands (dark green).

XYZ coordinates of the two DiRh/NiO interfaces

a-DiRh/NiO:V_{Ni}

Ni	25.073414	4.134881	4.109540
Ni	25.075407	20.883930	4.108272
Ni	25.077848	12.519682	4.107876
Ni	8.319649	4.127974	4.103398
Ni	8.316835	20.876650	4.107550
Ni	8.331091	12.500838	4.084114
Ni	16.699024	4.137038	4.098826
Ni	16.715614	20.873516	4.105259
Ni	16.691689	12.524023	4.085582
Ni	4.135558	16.688795	4.109952
Ni	4.142579	8.319355	4.095047
Ni	4.133221	25.068398	4.109205
Ni	12.498682	16.685312	4.070826
Ni	12.505318	8.451483	3.990237
Ni	12.510429	25.068726	4.102396
Ni	20.876839	16.760729	4.271278
Ni	20.891352	8.328330	4.106528
Ni	20.887970	25.071764	4.109223
Ni	2.040493	2.039039	4.109178
Ni	2.042427	18.786755	4.110051
Ni	2.054780	10.417680	4.108050
Ni	10.411635	2.038476	4.105524
Ni	10.404261	18.786766	4.093108
Ni	10.498715	10.434136	4.042777
Ni	18.794897	2.040159	4.108919
Ni	18.861715	18.819588	4.197219
Ni	18.816462	10.434709	4.100675
Ni	6.231205	6.228915	4.098617
Ni	6.225747	22.972633	4.109561
Ni	6.234326	14.592340	4.109699
Ni	14.612091	6.245521	4.175639
Ni	14.604717	22.967495	4.105701
Ni	14.607063	14.601743	4.107352
Ni	22.982813	6.231755	4.110636
Ni	22.982096	22.978704	4.107790
Ni	22.966145	14.625924	4.112048
Ni	4.133652	4.132285	4.108612
Ni	4.133739	20.878899	4.109719
Ni	4.159641	12.497040	4.107429
Ni	12.516626	4.127875	4.086311
Ni	12.513894	20.887339	4.213693
Ni	12.493393	12.434363	4.046258
Ni	20.888935	4.135801	4.110209
Ni	20.890535	20.889454	4.101587
Ni	20.889492	12.537422	4.110297
Ni	2.042698	6.228264	4.109139
Ni	2.041522	22.975513	4.108890

Ni	2.046756	14.599046	4.109504
Ni	10.413319	6.243271	4.071267
Ni	10.414989	22.966764	4.099104
Ni	10.423330	14.589623	4.104109
Ni	18.800312	6.234181	4.108417
Ni	18.795004	22.981283	4.104889
Ni	18.803417	14.630759	4.106820
Ni	25.073055	16.697220	4.110816
Ni	25.077436	8.325654	4.108728
Ni	25.073393	25.070929	4.108843
Ni	8.323125	16.688643	4.112684
Ni	8.280756	8.292358	4.142393
Ni	8.319452	25.066105	4.109158
Ni	16.712572	16.701326	4.109651
Ni	16.708204	8.341181	4.063326
Ni	16.699385	25.066998	4.108374
Ni	6.225221	2.036376	4.110324
Ni	6.225812	18.781933	4.110417
Ni	6.250229	10.368088	4.250273
Ni	14.609577	2.038626	4.099980
Ni	14.626817	18.793207	4.081064
Ni	14.477434	10.438069	3.957033
Ni	22.980623	2.040599	4.109314
Ni	22.987225	18.791691	4.100710
Ni	22.986504	10.427664	4.108360
O	2.050812	16.698038	4.115743
O	2.044496	8.327382	4.112547
O	2.048915	25.079309	4.114864
O	10.418161	16.699322	4.112496
O	10.466366	8.319247	4.033942
O	10.426606	25.079203	4.119995
O	18.746428	16.661306	4.010213
O	18.795053	8.344171	4.111177
O	18.797405	25.085382	4.117763
O	2.042052	4.137256	4.113844
O	2.054117	20.893488	4.115587
O	2.057861	12.511194	4.112104
O	10.435172	4.143423	4.108864
O	10.384762	20.883497	4.098106
O	10.430303	12.518906	4.081120
O	18.797611	4.145411	4.120946
O	18.801224	20.940168	4087869
O	18.785753	12.515198	.117517
O	6.236170	16.693871	4.118710
O	6.174147	8.270135	4000413
O	6.235600	25.076889	4.116949
O	14.603713	16.700439	4.126319
O	14.602733	8.358374	4.003441
O	14.608574	25.085047	4.118847
O	23.017963	16.706842	4.110948
O	22.985870	8.339330	4.115432
O	22.988710	25.084141	4.115033
O	6.229469	4.130788	4.113742

O	6.236386	20.887035	4.118368
O	6.249921	12.539169	4.096250
O	14.617808	4.109513	4.082480
O	14.630741	20.894238	4.106662
O	14.613712	12.516866	4.080822
O	22.983494	4.144819	4.115786
O	23.002577	20.907276	4.109135
O	22.991877	12.512120	4.111618
O	25.078062	2.048689	4.114806
O	25.091970	18.803135	4.115327
O	25.085932	10.425230	4.100161
O	8.332937	2.042922	4.117511
O	8.324852	18.785603	4.117300
O	8.373336	10.456854	3.904032
O	16.709148	2.051228	4.120672
O	16.666132	18.804697	4.093048
O	16.721949	10.434498	3.955717
O	25.075693	6.235726	4.114702
O	25.086241	22.990938	4.114269
O	25.081791	14.604291	4.113548
O	8.338611	6.177442	4.065096
O	8.330540	22.987186	4.120235
O	8.345916	14.603537	4.118422
O	16.738113	6.252920	4.094240
O	16.699406	22.991756	4.115120
O	16.691299	14.593605	4.104846
O	4.138374	2.043128	4.114928
O	4.142797	18.792395	4.116873
O	4.138534	10.416899	4.080497
O	12.514620	2.061441	4.097517
O	12.516638	18.776390	4.038571
O	12.527947	10.423271	4.323750
O	20.893106	2.051674	4.116705
O	20.959280	18.852684	4.027626
O	20.883474	10.428395	4.094655
O	4.127943	6.219510	4.105213
O	4.144564	22.984591	4.115787
O	4.141792	14.601435	4.112646
O	12.507920	6.215487	3.962193
O	12.512457	23.012306	4.092926
O	12.523974	14.619134	4.101734
O	20.892233	6.243621	4.117503
O	20.899744	22.997093	4.114320
O	20.894024	14.581075	4.096439
Ni	6.256377	16.721889	2.060121
Ni	6.250339	8.340377	2.023420
Ni	6.255640	25.095598	2.058526
Ni	14.635641	16.728277	2.059839
Ni	14.593758	8.377107	2.008831
Ni	14.634878	25.096409	2.058963
Ni	22.999834	16.729725	2.067082
Ni	23.009659	8.351473	2.058369
Ni	23.008062	25.101397	2.058155

Ni	25.100529	6.256596	2.058123
Ni	25.102274	23.006634	2.057827
Ni	25.101484	14.635912	2.059202
Ni	8.345452	6.241872	2.042535
Ni	8.348740	23.001778	2.058966
Ni	8.360338	14.632989	2.058539
Ni	16.728275	6.254953	2.055550
Ni	16.727152	23.004942	2.058573
Ni	16.724501	14.638410	2.054351
Ni	4.161502	2.065214	2.058177
Ni	4.163609	18.815912	2.058875
Ni	4.160905	10.436919	2.053484
Ni	12.534101	2.051596	2.050914
Ni	12.537521	18.827221	2.033930
Ni	20.915070	2.070326	2.058843
Ni	20.913916	18.812294	2.040223
Ni	20.933989	10.457365	2.052296
Ni	2.067806	4.160922	2.057928
Ni	2.071044	20.911394	2.058313
Ni	2.074708	12.540969	2.058719
Ni	10.435297	4.155033	2.053884
Ni	10.444848	20.909811	2.056658
Ni	10.497600	12.491777	2.036615
Ni	18.825054	4.163055	2.059988
Ni	18.822731	20.913708	2.055304
Ni	18.827032	12.554993	2.059560
Ni	2.070836	16.723913	2.059273
Ni	2.065988	8.347724	2.056496
Ni	2.069421	25.098875	2.058069
Ni	10.439850	16.721642	2.055245
Ni	10.471233	8.385036	2.027290
Ni	10.441489	25.094976	2.058933
Ni	18.827988	16.735523	2.034524
Ni	18.823402	8.344785	2.053145
Ni	18.821102	25.102634	2.059059
Ni	6.252453	4.152271	2.056585
Ni	6.255494	20.909002	2.058869
Ni	6.267056	12.534436	2.063394
Ni	14.646375	4.162304	2.050919
Ni	14.627558	20.911705	2.061418
Ni	14.581930	12.508376	2.031490
Ni	23.008034	4.163802	2.058595
Ni	23.009764	20.914145	2.055911
Ni	23.010847	12.548077	2.058775
Ni	25.100739	2.068824	2.058149
Ni	25.105341	18.818808	2.057858
Ni	25.105122	10.447450	2.054973
Ni	8.347945	2.059857	2.057981
Ni	8.347880	18.816034	2.057576
Ni	8.332977	10.437623	2.003597
Ni	16.728483	2.066528	2.059021
Ni	16.736635	18.821285	2.056703
Ni	16.753536	10.454023	2.008624

Ni	4.159106	6.251472	2.054650
Ni	4.163150	23.003796	2.058310
Ni	4.166549	14.627507	2.058405
Ni	12.542674	6.229929	2.016245
Ni	12.537343	22.999607	2.058358
Ni	12.541175	14.647129	2.055771
Ni	20.918062	6.259427	2.058821
Ni	20.915054	23.009796	2.057358
Ni	20.917248	14.651489	2.063116
O	2.068925	2.068416	2.058087
O	2.075479	18.819128	2.058721
O	2.069738	10.446690	2.055649
O	10.455233	2.070432	2.056583
O	10.436276	18.812386	2.050554
O	10.227915	10.442937	2.021681
O	18.820597	2.074759	2.058496
O	18.809311	18.829350	2.092159
O	18.703121	10.439712	2.043310
O	2.064256	6.254248	2.057538
O	2.073072	23.008139	2.058215
O	2.073571	14.629189	2.058098
O	10.402312	6.240207	2.032235
O	10.440997	23.010624	2.054635
O	10.438935	14.634651	2.055196
O	18.826925	6.258631	2.056305
O	18.820250	23.021671	2.054103
O	18.820084	14.617745	2.052141
O	6.256929	2.061511	2.059226
O	6.256978	18.816275	2.059564
O	6.368567	10.446431	2.066916
O	14.625266	2.068913	2.051117
O	14.626814	18.817869	2.043536
O	14.871268	10.453685	2.004759
O	23.007425	2.072984	2.058453
O	23.027437	18.824993	2.051118
O	23.010420	10.442257	2.056449
O	6.248447	6.225695	2.047550
O	6.259119	23.005480	2.059377
O	6.257841	14.634136	2.056453
O	14.673724	6.241574	2.082962
O	14.633117	23.010984	2.057767
O	14.642950	14.631737	2.056118
O	23.005758	6.260773	2.059363
O	23.011959	23.014074	2.057329
O	23.012329	14.629924	2.060115
O	25.107475	16.725361	2.057512
O	25.098339	8.350968	2.057818
O	25.102755	25.103409	2.058023
O	8.348253	16.717930	2.059674
O	8.358867	8.283765	2.069358
O	8.353796	25.098171	2.059059
O	16.705498	16.714487	2.055794
O	16.734093	8.304027	2.034441

O	16.725109	25.106138	2.058458
O	25.099014	4.164219	2.058419
O	25.108377	20.917191	2.057630
O	25.103926	12.537738	2.057065
O	8.349185	4.142678	2.051893
O	8.345005	20.911467	2.055805
O	8.362303	12.596718	2.041516
O	16.737436	4.163825	2.054116
O	16.722532	20.921772	2.054779
O	16.728975	12.591298	2.043338
O	4.165653	16.723036	2.058944
O	4.143558	8.350695	2.045476
O	4.164596	25.099024	2.058401
O	12.537768	16.686869	2.040418
O	12.539733	8.116041	2.016129
O	12.541595	25.103413	2.053829
O	20.921946	16.716789	2.097331
O	20.908875	8.363586	2.057085
O	20.914856	25.108295	2.058360
O	4.159040	4.155591	2.057112
O	4.165931	20.912182	2.059083
O	4.171931	12.527690	2.057036
O	12.542501	4.271303	2.035734
O	12.529551	20.922501	2.099816
O	12.540647	12.693951	2.042734
O	20.914309	4.167183	2.059482
O	20.921471	20.930367	2.051021
O	20.910549	12.524193	2.055407
Ni	25.125401	16.750437	0.009081
Ni	25.125372	8.375380	0.009112
Ni	25.125422	25.125401	0.009080
Ni	8.375393	16.750471	0.009094
Ni	8.375295	8.375462	0.009181
Ni	8.375384	25.125452	0.009064
Ni	16.750525	16.750450	0.009033
Ni	16.750422	8.375485	0.009011
Ni	16.750381	25.125427	0.009043
Ni	6.281582	2.094148	0.009116
Ni	6.281607	18.844110	0.009033
Ni	6.281603	10.469150	0.009112
Ni	14.656700	2.094160	0.009160
Ni	14.656680	18.844078	0.009003
Ni	14.656655	10.469081	0.009094
Ni	23.031750	2.094094	0.009131
Ni	23.031691	18.844170	0.009040
Ni	23.031660	10.469163	0.009067
Ni	4.187876	4.187797	0.009068
Ni	4.187904	20.937922	0.009074
Ni	4.187919	12.562900	0.009121
Ni	12.562885	4.187901	0.009032
Ni	12.562889	20.937897	0.008999
Ni	12.562933	12.562975	0.009074
Ni	20.937918	4.187825	0.009156

Ni	20.937853	20.938004	0.009077
Ni	20.937893	12.562897	0.009061
Ni	2.094159	6.281636	0.009046
Ni	2.094081	23.031601	0.009022
Ni	2.094155	14.656665	0.009094
Ni	10.469164	6.281599	0.008980
Ni	10.469177	23.031630	0.009027
Ni	10.469124	14.656572	0.009039
Ni	18.844212	6.281625	0.009109
Ni	18.844215	23.031590	0.009063
Ni	18.844093	14.656574	0.009066
Ni	6.281665	6.281707	0.009054
Ni	6.281603	23.031658	0.009137
Ni	6.281638	14.656595	0.009112
Ni	14.656655	6.281608	0.009127
Ni	14.656664	23.031687	0.009078
Ni	14.656587	14.656571	0.009048
Ni	23.031694	6.281626	0.009105
Ni	23.031736	23.031679	0.009105
Ni	23.031643	14.656649	0.009061
Ni	25.125427	4.187800	0.009149
Ni	25.125368	20.937935	0.009044
Ni	25.125435	12.562939	0.009058
Ni	8.375406	4.187928	0.008965
Ni	8.375410	20.937927	0.009089
Ni	8.375406	12.562954	0.009124
Ni	16.750383	4.187917	0.009153
Ni	16.750397	20.937916	0.009128
Ni	16.750414	12.562978	0.009091
Ni	4.187833	16.750465	0.009000
Ni	4.187726	8.375332	0.009046
Ni	4.187963	25.125399	0.009021
Ni	12.562967	16.750441	0.008999
Ni	12.562879	8.375468	0.009079
Ni	12.562897	25.125401	0.009082
Ni	20.937979	16.750481	0.009142
Ni	20.937965	8.375315	0.009086
Ni	20.937815	25.125435	0.009050
Ni	2.094118	2.094085	0.009136
Ni	2.094135	18.844175	0.009040
Ni	2.094053	10.469115	0.009069
Ni	10.469122	2.094080	0.008989
Ni	10.469105	18.844118	0.008964
Ni	10.469141	10.469044	0.009080
Ni	18.844263	2.094087	0.009090
Ni	18.844177	18.844048	0.009173
Ni	18.844198	10.469126	0.009094
O	2.093840	16.750187	0.000000
O	2.093770	8.375137	0.000000
O	2.093965	25.125063	0.000000
O	10.468937	16.750111	0.000000
O	10.468853	8.375086	0.000000
O	10.468903	25.125063	0.000000

O	18.844006	16.750080	0.000000
O	18.843969	8.375148	0.000000
O	18.843857	25.125063	0.000000
O	2.093883	4.187632	0.000000
O	2.093872	20.937658	0.000000
O	2.093920	12.562648	0.000000
O	10.468899	4.187603	0.000000
O	10.468901	20.937639	0.000000
O	10.468930	12.562567	0.000000
O	18.843899	4.187607	0.000000
O	18.843880	20.937576	0.000000
O	18.843922	12.562605	0.000000
O	6.281359	16.750122	0.000000
O	6.281225	8.375201	0.000000
O	6.281418	25.125063	-0.000458
O	14.656547	16.750057	0.000000
O	14.656427	8.375104	0.000000
O	14.656396	25.125063	0.000000
O	23.031445	16.750175	0.000000
O	23.031437	8.375166	0.000000
O	23.031368	25.125063	0.000000
O	6.281430	4.187720	0.000000
O	6.281423	20.937632	0.000000
O	6.281417	12.562621	0.000000
O	14.656403	4.187659	0.000000
O	14.656406	20.937654	0.000000
O	14.656434	12.562582	0.000000
O	23.031431	4.187614	0.000000
O	23.031372	20.937691	0.000000
O	23.031424	12.562651	0.000000
O	25.125063	2.093787	0.000000
O	25.125063	18.843987	0.000000
O	25.125063	10.468916	0.000000
O	8.375102	2.093923	0.000000
O	8.375106	18.843950	0.000000
O	8.375123	10.468974	0.000000
O	16.750252	2.093898	0.000000
O	16.750177	18.843948	0.000000
O	16.750193	10.468990	0.000000
O	25.125063	6.281361	0.000000
O	25.125063	23.031424	0.000000
O	25.125063	14.656451	0.000000
O	8.375178	6.281464	0.000000
O	8.375149	23.031431	0.000000
O	8.375146	14.656481	0.000000
O	16.750202	6.281458	0.000000
O	16.750196	23.031420	0.000000
O	16.750086	14.656476	0.000000
O	4.187592	2.093828	0.000000
O	4.187634	18.843960	0.000000
O	4.187577	10.468869	0.000000
O	12.562663	2.093871	0.000000
O	12.562648	18.843933	0.000000

O	12.562662	10.469010	0.000000
O	20.937780	2.093838	0.000000
O	20.937702	18.844076	0.000000
O	20.937706	10.468858	0.000000
O	4.187682	6.281311	0.000000
O	4.187583	23.031418	0.000000
O	4.187675	14.656466	0.000000
O	12.562665	6.281444	0.000000
O	12.562681	23.031389	0.000000
O	12.562609	14.656481	0.000000
O	20.937717	6.281321	0.000000
O	20.937756	23.031485	0.000000
O	20.937601	14.656458	0.000000
Rh	13.917899	14.556839	10.443990
Rh	13.154680	12.265025	10.307461
N	14.611769	16.634077	10.506446
N	12.500870	10.183589	10.167291
C	14.954831	17.662033	10.078027
C	15.368210	18.919134	9.492477
C	12.217188	9.140730	9.732389
C	11.863271	7.863310	9.150239
H	15.451987	19.696613	10.262899
H	14.624484	19.228222	8.745214
H	16.337561	18.788254	8.991475
H	11.918253	7.067615	9.904681
H	12.558990	7.632926	8.332027
H	10.844551	7.915604	8.741600
N	12.014233	12.821644	8.635651
N	12.527156	15.092901	8.987013
C	11.887040	14.115449	8.358757
C	10.966216	12.007499	8.133944
C	9.630084	12.338821	8.429075
C	8.585231	11.566553	7.932011
C	8.866696	10.413832	7.191761
C	7.798423	9.636603	6.488873
C	10.203910	10.067602	6.921651
C	11.247959	10.873103	7.364157
C	12.264992	16.418888	8.576476
C	11.690874	17.342678	9.475627
C	11.499550	18.669432	9.104670
C	11.921295	19.112661	7.833303
C	11.834992	20.516253	7.392224
C	12.477174	18.187977	6.939122
C	12.612130	16.851774	7.288654
O	6.716750	10.259258	6.219450
O	8.128121	8.458473	6.119193
O	11.163964	21.327095	8.253363
O	12.329258	20.980291	6.362234
H	11.175360	14.392563	7.568461
H	9.426831	13.204317	9.061208
H	7.545284	11.842446	8.108370
H	10.398312	9.168620	6.332382
H	12.274813	10.643777	7.087458

H	11.397926	17.002684	10.468093
H	11.033272	19.366966	9.800164
H	11.164331	22.211052	7.830228
H	12.782671	18.529396	5.948849
H	13.022766	16.135443	6.572637
N	14.790284	11.846448	9.097780
N	15.332511	14.129138	8.951408
C	15.506794	12.854964	8.621386
C	15.073309	10.542615	8.638600
C	15.558715	9.562962	9.530104
C	15.713343	8.244484	9.115621
C	15.342286	7.875972	7.806028
C	15.331277	6.483292	7.330416
C	14.892971	8.861283	6.915503
C	14.793873	10.186980	7.312397
C	16.330622	15.012465	8.451766
C	17.677784	14.836360	8.817528
C	18.673813	15.622486	8.238358
C	18.331236	16.649090	7.350250
C	19.344830	17.427319	6.559142
C	16.976250	16.864437	7.038480
C	15.987781	16.033682	7.557200
O	15.967021	5.605508	8.149365
O	14.771508	6.082483	6.305855
O	20.427679	16.824083	6.258851
O	18.958403	18.580860	6.170160
H	16.313658	12.620231	7.913349
H	15.791591	9.849979	10.554910
H	16.094948	7.494638	9.808482
H	15.890725	4.734073	7.706734
H	14.614534	8.574967	5.899676
H	14.445195	10.951026	6.612703
H	17.932442	14.064073	9.545262
H	19.726025	15.442577	8.461668
H	16.721024	17.686390	6.361890
H	14.952368	16.157141	7.251849
N	12.326398	14.391168	11.746576
N	11.791693	13.177301	11.498744
C	11.744841	15.250543	12.646016
C	10.500909	14.930418	13.272049
C	9.947424	15.863069	14.167669
C	10.588749	17.064795	14.453191
C	11.824240	17.357901	13.850243
C	9.889472	13.652646	12.947204
C	8.664124	13.206373	13.477577
C	8.137866	11.962133	13.144888
C	8.835115	11.126356	12.254765
C	10.043241	11.531427	11.714599
C	10.592285	12.792377	12.049661
C	12.400608	16.464855	12.960837
H	8.997552	15.641074	14.653072
H	10.136029	17.769533	15.150707
H	12.339883	18.289589	14.086689

H	8.112316	13.845599	14.166181
H	7.186813	11.640581	13.569788
H	8.421565	10.156066	11.975920
H	10.578036	10.899579	11.013834
H	13.360813	16.671095	12.493256
N	15.061100	13.541373	11.780832
N	14.488732	12.322445	11.870019
C	16.184362	13.857686	12.504915
C	16.747486	12.925188	13.429296
C	17.895332	13.309045	14.146426
C	18.478327	14.560010	13.970985
C	17.921000	15.465235	13.051243
C	16.082369	11.642193	13.584563
C	16.501158	10.639250	14.477599
C	15.814284	9.433871	14.589802
C	14.665794	9.209665	13.810695
C	14.223234	10.173484	12.918833
C	14.930410	11.392025	12.777765
C	16.792988	15.122541	12.326214
H	18.339958	12.614599	14.858630
H	19.365999	14.832962	14.541827
H	18.379915	16.443087	12.898176
H	17.381046	10.806653	15.097830
H	16.162203	8.672512	15.287951
H	14.111488	8.275381	13.911090
H	13.331324	10.019557	12.317038
H	16.362190	15.809525	11.604733

s-DiRh/NiO:V_{Ni}

Ni	25.060469	4.145147	4.108407
Ni	25.080463	20.891359	4.109073
Ni	25.078293	12.516334	4.108126
Ni	8.327963	4.138241	4.109649
Ni	8.338997	20.872517	4.110056
Ni	8.349476	12.518001	4.080305
Ni	16.713356	4.180595	4.112576
Ni	16.704542	20.902235	4.112723
Ni	16.696121	12.527247	4.093575
Ni	4.162802	16.701916	4.113826
Ni	4.134705	8.319145	4.105954
Ni	4.137156	25.072611	4.108650
Ni	12.549239	16.711403	4.082147
Ni	12.525792	8.429130	4.041669
Ni	12.515221	25.079149	4.108268
Ni	20.897921	16.711113	4.111599
Ni	20.840698	8.346290	4.272715
Ni	20.883802	25.093552	4.109505
Ni	2.037598	2.045472	4.108679
Ni	2.056826	18.794424	4.109673
Ni	2.041919	10.419944	4.106580
Ni	10.419485	2.044853	4.108147
Ni	10.427812	18.770124	4.247325

Ni	10.551363	10.429808	4.024898
Ni	18.790440	2.076213	4.115010
Ni	18.802172	18.807831	4.112013
Ni	18.805624	10.462602	4.078935
Ni	6.233461	6.229498	4.108337
Ni	6.236492	22.973902	4.108154
Ni	6.268957	14.626443	4.112519
Ni	14.599380	6.255302	4.094996
Ni	14.609107	22.988848	4.110404
Ni	14.617760	14.606561	4.096792
Ni	22.953918	6.250217	4.113447
Ni	22.981152	22.990871	4.109055
Ni	22.991169	14.612948	4.110568
Ni	4.132606	4.135845	4.108947
Ni	4.148622	20.882524	4.108544
Ni	4.150119	12.523426	4.106017
Ni	12.516955	4.142259	4.108112
Ni	12.509858	20.877281	4.108972
Ni	12.518804	12.417561	4.032221
Ni	20.860668	4.175650	4.113423
Ni	20.891518	20.899580	4.109899
Ni	20.890799	12.531828	4.106529
Ni	2.032799	6.230750	4.109038
Ni	2.046035	22.981506	4.108698
Ni	2.057203	14.610676	4.110382
Ni	10.435025	6.245836	4.093432
Ni	10.422112	22.974882	4.108238
Ni	10.446885	14.590508	4.060134
Ni	18.796906	6.362399	4.319056
Ni	18.795206	22.997894	4.110379
Ni	18.801628	14.621635	4.117119
Ni	25.087275	16.704309	4.110007
Ni	25.060192	8.327327	4.109204
Ni	25.070841	25.081228	4.108683
Ni	8.428470	16.723692	4.293488
Ni	8.342568	8.329339	4.077219
Ni	8.326357	25.070023	4.108810
Ni	16.712517	16.711435	4.116683
Ni	16.678783	8.354703	4.055231
Ni	16.702084	25.092831	4.110284
Ni	6.230442	2.040770	4.108896
Ni	6.260028	18.778662	4.109828
Ni	6.224193	10.419641	4.089233
Ni	14.613519	2.059208	4.108475
Ni	14.623329	18.801657	4.106142
Ni	14.510340	10.421160	4.031023
Ni	22.969748	2.060203	4.108717
Ni	22.989546	18.800974	4.109738
Ni	22.964087	10.416613	4.107801
O	2.062785	16.704504	4.114549
O	2.049245	8.339260	4.114401
O	2.048509	25.082907	4.114481
O	10.518641	16.630037	3.966205

O	10.426665	8.346275	4.066611
O	10.427810	25.081074	4.115389
O	18.807508	16.716105	4.125637
O	18.719942	8.438375	3.959406
O	18.798285	25.097502	4.114146
O	2.047065	4.147222	4.114936
O	2.053586	20.894630	4.113972
O	2.058445	12.514595	4.112829
O	10.420350	4.149524	4.114705
O	10.441895	20.915102	4.112322
O	10.448694	12.510903	4.074443
O	18.783813	4.150490	4.127325
O	18.802788	20.903099	4.116098
O	18.789520	12.546040	4.115996
O	6.233928	16.695160	4.120163
O	6.248311	8.336488	4.110330
O	6.237604	25.080194	4.114697
O	14.636321	16.699001	4.117286
O	14.605141	8.359756	4.084211
O	14.611415	25.088623	4.112856
O	22.995625	16.710045	4.116150
O	23.002382	8.353599	4.120130
O	22.985649	25.089058	4.113986
O	6.238072	4.145660	4.116334
O	6.241633	20.893875	4.111313
O	6.246953	12.514253	4.105872
O	14.614157	4.149961	4.111440
O	14.615590	20.902172	4.112476
O	14.624800	12.535262	4.084592
O	22.981987	4.155302	4.112520
O	22.990047	20.898226	4.114823
O	22.995884	12.523818	4.113118
O	25.077791	2.055541	4.114221
O	25.088230	18.801548	4.114763
O	25.082869	10.434559	4.099523
O	8.330426	2.049636	4.115354
O	8.315093	18.819803	4.078641
O	8.323363	10.433965	3.967633
O	16.698900	2.062638	4.112484
O	16.713066	18.808779	4.123270
O	16.702444	10.466334	3.950645
O	25.075253	6.243263	4.114824
O	25.081337	22.990585	4.114309
O	25.090618	14.614669	4.113785
O	8.337852	6.246169	4.114208
O	8.333196	22.984674	4.114324
O	8.352290	14.564532	4.080428
O	16.654301	6.265578	4.082652
O	16.708570	22.997475	4.112103
O	16.713787	14.624138	4.130585
O	4.142123	2.050520	4.115047
O	4.152570	18.801693	4.114132
O	4.160796	10.423723	4.093611

O	12.516920	2.057699	4.111450
O	12.579123	18.787271	4.063722
O	12.532638	10.440166	4.347951
O	20.892651	2.065554	4.114507
O	20.899321	18.806999	4.117264
O	20.879692	10.490911	4.059562
O	4.143192	6.240788	4.115715
O	4.144889	22.987162	4.113981
O	4.154788	14.604069	4.111792
O	12.520270	6.228166	4.075228
O	12.526138	22.989723	4.102837
O	12.554310	14.609035	3.950477
O	20.900473	6.236714	4.098793
O	20.893909	22.996969	4.114589
O	20.901133	14.621785	4.121084
Ni	6.277843	16.732283	2.070737
Ni	6.256230	8.340797	2.054615
Ni	6.258660	25.100061	2.058459
Ni	14.655888	16.734459	2.058241
Ni	14.576247	8.402656	2.031957
Ni	14.635449	25.107885	2.058555
Ni	23.014460	16.731766	2.058928
Ni	22.991379	8.351444	2.071292
Ni	23.006353	25.108213	2.058304
Ni	25.093086	6.260985	2.059579
Ni	25.102476	23.010910	2.058159
Ni	25.107656	14.636956	2.058468
Ni	8.354809	6.254583	2.056309
Ni	8.354487	23.004784	2.059024
Ni	8.360493	14.643504	2.054281
Ni	16.728594	6.268949	2.055592
Ni	16.729399	23.016439	2.058249
Ni	16.739014	14.648144	2.060952
Ni	4.162459	2.069744	2.058567
Ni	4.171825	18.820127	2.058919
Ni	4.147965	10.439230	2.050104
Ni	12.539315	2.071247	2.056954
Ni	12.540491	18.838583	2.052066
Ni	20.910591	2.082708	2.059616
Ni	20.919653	18.827776	2.059098
Ni	20.929667	10.445298	2.050632
Ni	2.065724	4.164976	2.058685
Ni	2.074174	20.915075	2.058192
Ni	2.073821	12.543944	2.058758
Ni	10.442288	4.165089	2.057904
Ni	10.445226	20.904474	2.067463
Ni	10.490776	12.496192	2.029744
Ni	18.821533	4.191743	2.074441
Ni	18.823944	20.922945	2.059146
Ni	18.825901	12.564345	2.057000
Ni	2.079107	16.729322	2.059240
Ni	2.064724	8.348320	2.058971
Ni	2.069624	25.102791	2.058273

Ni	10.438419	16.728893	2.015602
Ni	10.498591	8.393002	2.035003
Ni	10.446007	25.101360	2.059173
Ni	18.830786	16.738968	2.062643
Ni	18.819120	8.344906	2.013054
Ni	18.821230	25.114086	2.059305
Ni	6.255563	4.162035	2.058946
Ni	6.264200	20.910784	2.058447
Ni	6.264795	12.553031	2.056374
Ni	14.638857	4.172549	2.058671
Ni	14.638690	20.919704	2.058928
Ni	14.602468	12.508224	2.037328
Ni	22.999596	4.173420	2.059483
Ni	23.010689	20.919008	2.058301
Ni	23.010862	12.545888	2.058952
Ni	25.097956	2.074405	2.058454
Ni	25.107027	18.823090	2.058328
Ni	25.099121	10.446513	2.055422
Ni	8.351585	2.069139	2.058715
Ni	8.365886	18.807598	2.063058
Ni	8.335062	10.445034	2.016716
Ni	16.729568	2.082224	2.059504
Ni	16.734806	18.830153	2.061094
Ni	16.739389	10.459588	2.008544
Ni	4.159774	6.255500	2.058574
Ni	4.166310	23.007107	2.058261
Ni	4.171703	14.637281	2.059053
Ni	12.539338	6.241053	2.043317
Ni	12.540512	23.008171	2.055666
Ni	12.553393	14.646366	2.008915
Ni	20.894009	6.277032	2.072200
Ni	20.914988	23.015928	2.058380
Ni	20.921530	14.642365	2.060384
O	2.070008	2.072931	2.058616
O	2.073813	18.821222	2.058898
O	2.069976	10.449918	2.057193
O	10.447315	2.074209	2.058353
O	10.458736	18.820757	2.096567
O	10.222199	10.445816	2.031767
O	18.818844	2.081369	2.060441
O	18.825518	18.826647	2.060065
O	18.687698	10.484119	2.029496
O	2.070922	6.261965	2.059115
O	2.071554	23.009680	2.058422
O	2.076598	14.631811	2.058799
O	10.426842	6.260524	2.048628
O	10.448047	23.010197	2.056275
O	10.414936	14.604518	2.028058
O	18.807983	6.250506	2.096364
O	18.821692	23.014044	2.059149
O	18.825569	14.650227	2.061489
O	6.258115	2.072140	2.058772
O	6.256610	18.820696	2.057356

O	6.378331	10.449886	2.037496
O	14.629138	2.077119	2.057331
O	14.639501	18.817808	2.052387
O	14.851098	10.461251	2.025816
O	23.007614	2.076241	2.058386
O	23.011478	18.823500	2.059016
O	23.013214	10.452444	2.056382
O	6.252064	6.255542	2.058612
O	6.260176	23.008989	2.058019
O	6.259836	14.630263	2.059011
O	14.636539	6.262470	2.046584
O	14.636157	23.011209	2.058214
O	14.697354	14.637971	2.048706
O	23.006323	6.266749	2.060496
O	23.008902	23.011736	2.058681
O	23.011616	14.636816	2.058945
O	25.106592	16.727907	2.059030
O	25.101793	8.356661	2.057075
O	25.101915	25.104868	2.058510
O	8.338576	16.710020	2.098452
O	8.347986	8.301526	2.038875
O	8.353309	25.103046	2.058600
O	16.741537	16.734192	2.061241
O	16.692984	8.319902	2.026569
O	16.726723	25.108347	2.058676
O	25.100632	4.169269	2.058429
O	25.103823	20.916231	2.058636
O	25.105543	12.543090	2.057233
O	8.348639	4.169217	2.059089
O	8.356598	20.917259	2.057580
O	8.355122	12.579177	2.038001
O	16.724209	4.174467	2.059507
O	16.729738	20.917835	2.059806
O	16.729454	12.607999	2.047481
O	4.169755	16.725235	2.059532
O	4.169606	8.361457	2.057026
O	4.164881	25.103224	2.058456
O	12.573624	16.592873	2.031615
O	12.539364	8.197503	2.042481
O	12.540752	25.097097	2.058003
O	20.919437	16.733034	2.059829
O	20.913527	8.366013	2.097708
O	20.914322	25.108040	2.058893
O	4.163218	4.165421	2.058981
O	4.166589	20.915325	2.058045
O	4.174111	12.526543	2.055496
O	12.537562	4.191632	2.056656
O	12.544352	20.917412	2.056957
O	12.552594	12.757542	2.025923
O	20.912336	4.170203	2.060418
O	20.916460	20.918837	2.059092
O	20.911629	12.547631	2.052401
Ni	25.125401	16.750437	0.009081

Ni	25.125372	8.375380	0.009112
Ni	25.125422	25.125401	0.009080
Ni	8.375393	16.750471	0.009094
Ni	8.375295	8.375462	0.009181
Ni	8.375384	25.125452	0.009064
Ni	16.750525	16.750450	0.009033
Ni	16.750422	8.375485	0.009011
Ni	16.750381	25.125427	0.009043
Ni	6.281582	2.094148	0.009116
Ni	6.281607	18.844110	0.009033
Ni	6.281603	10.469150	0.009112
Ni	14.656700	2.094160	0.009160
Ni	14.656680	18.844078	0.009003
Ni	14.656655	10.469081	0.009094
Ni	23.031750	2.094094	0.009131
Ni	23.031691	18.844170	0.009040
Ni	23.031660	10.469163	0.009067
Ni	4.187876	4.187797	0.009068
Ni	4.187904	20.937922	0.009074
Ni	4.187919	12.562900	0.009121
Ni	12.562885	4.187901	0.009032
Ni	12.562889	20.937897	0.008999
Ni	12.562933	12.562975	0.009074
Ni	20.937918	4.187825	0.009156
Ni	20.937853	20.938004	0.009077
Ni	20.937893	12.562897	0.009061
Ni	2.094159	6.281636	0.009046
Ni	2.094081	23.031601	0.009022
Ni	2.094155	14.656665	0.009094
Ni	10.469164	6.281599	0.008980
Ni	10.469177	23.031630	0.009027
Ni	10.469124	14.656572	0.009039
Ni	18.844212	6.281625	0.009109
Ni	18.844215	23.031590	0.009063
Ni	18.844093	14.656574	0.009066
Ni	6.281665	6.281707	0.009054
Ni	6.281603	23.031658	0.009137
Ni	6.281638	14.656595	0.009112
Ni	14.656655	6.281608	0.009127
Ni	14.656664	23.031687	0.009078
Ni	14.656587	14.656571	0.009048
Ni	23.031694	6.281626	0.009105
Ni	23.031736	23.031679	0.009105
Ni	23.031643	14.656649	0.009061
Ni	25.125427	4.187800	0.009149
Ni	25.125368	20.937935	0.009044
Ni	25.125435	12.562939	0.009058
Ni	8.375406	4.187928	0.008965
Ni	8.375410	20.937927	0.009089
Ni	8.375406	12.562954	0.009124
Ni	16.750383	4.187917	0.009153
Ni	16.750397	20.937916	0.009128
Ni	16.750414	12.562978	0.009091

Ni	4.187833	16.750465	0.009000
Ni	4.187726	8.375332	0.009046
Ni	4.187963	25.125399	0.009021
Ni	12.562967	16.750441	0.008999
Ni	12.562879	8.375468	0.009079
Ni	12.562897	25.125401	0.009082
Ni	20.937979	16.750481	0.009142
Ni	20.937965	8.375315	0.009086
Ni	20.937815	25.125435	0.009050
Ni	2.094118	2.094085	0.009136
Ni	2.094135	18.844175	0.009040
Ni	2.094053	10.469115	0.009069
Ni	10.469122	2.094080	0.008989
Ni	10.469105	18.844118	0.008964
Ni	10.469141	10.469044	0.009080
Ni	18.844263	2.094087	0.009090
Ni	18.844177	18.844048	0.009173
Ni	18.844198	10.469126	0.009094
O	2.093840	16.750187	0.000000
O	2.093770	8.375137	0.000000
O	2.093965	25.125063	0.000000
O	10.468937	16.750111	0.000000
O	10.468853	8.375086	0.000000
O	10.468903	25.125063	0.000000
O	18.844006	16.750080	0.000000
O	18.843969	8.375148	0.000000
O	18.843857	25.125063	0.000000
O	2.093883	4.187632	0.000000
O	2.093872	20.937658	0.000000
O	2.093920	12.562648	0.000000
O	10.468899	4.187603	0.000000
O	10.468901	20.937639	0.000000
O	10.468930	12.562567	0.000000
O	18.843899	4.187607	0.000000
O	18.843880	20.937576	0.000000
O	18.843922	12.562605	0.000000
O	6.281359	16.750122	0.000000
O	6.281225	8.375201	0.000000
O	6.281418	25.125063	-0.000458
O	14.656547	16.750057	0.000000
O	14.656427	8.375104	0.000000
O	14.656396	25.125063	0.000000
O	23.031445	16.750175	0.000000
O	23.031437	8.375166	0.000000
O	23.031368	25.125063	0.000000
O	6.281430	4.187720	0.000000
O	6.281423	20.937632	0.000000
O	6.281417	12.562621	0.000000
O	14.656403	4.187659	0.000000
O	14.656406	20.937654	0.000000
O	14.656434	12.562582	0.000000
O	23.031431	4.187614	0.000000
O	23.031372	20.937691	0.000000

O	23.031424	12.562651	0.000000
O	25.125063	2.093787	0.000000
O	25.125063	18.843987	0.000000
O	25.125063	10.468916	0.000000
O	8.375102	2.093923	0.000000
O	8.375106	18.843950	0.000000
O	8.375123	10.468974	0.000000
O	16.750252	2.093898	0.000000
O	16.750177	18.843948	0.000000
O	16.750193	10.468990	0.000000
O	25.125063	6.281361	0.000000
O	25.125063	23.031424	0.000000
O	25.125063	14.656451	0.000000
O	8.375178	6.281464	0.000000
O	8.375149	23.031431	0.000000
O	8.375146	14.656481	0.000000
O	16.750202	6.281458	0.000000
O	16.750196	23.031420	0.000000
O	16.750086	14.656476	0.000000
O	4.187592	2.093828	0.000000
O	4.187634	18.843960	0.000000
O	4.187577	10.468869	0.000000
O	12.562663	2.093871	0.000000
O	12.562648	18.843933	0.000000
O	12.562662	10.469010	0.000000
O	20.937780	2.093838	0.000000
O	20.937702	18.844076	0.000000
O	20.937706	10.468858	0.000000
O	4.187682	6.281311	0.000000
O	4.187583	23.031418	0.000000
O	4.187675	14.656466	0.000000
O	12.562665	6.281444	0.000000
O	12.562681	23.031389	0.000000
O	12.562609	14.656481	0.000000
O	20.937717	6.281321	0.000000
O	20.937756	23.031485	0.000000
O	20.937601	14.656458	0.000000
Rh	15.712162	12.148305	11.291525
Rh	14.033496	13.892414	11.195824
N	17.244188	10.602807	11.369016
N	12.516195	15.469377	11.053774
C	18.236694	10.011786	11.221300
C	19.486624	9.308502	11.036696
C	11.928083	16.425907	10.744679
C	11.227039	17.630056	10.356333
H	19.547583	8.451192	11.719987
H	20.318361	9.995903	11.245310
H	19.559837	8.956362	9.999094
H	10.778489	18.109730	11.236386
H	10.443652	17.389429	9.625854
H	11.938283	18.324442	9.888754
N	15.939270	12.261861	9.191815
N	14.218756	13.874629	9.101309

C	15.081256	13.038662	8.529633
C	13.286196	14.611446	8.301114
C	11.991947	14.103392	8.080975
C	10.998690	14.903953	7.513804
C	11.267619	16.234480	7.143992
C	12.586446	16.709896	7.282046
C	10.199950	17.111361	6.509887
C	13.582456	15.909329	7.850660
C	16.713198	11.301533	8.458087
C	16.122143	10.130810	7.946022
C	16.905844	9.120364	7.378810
C	18.301573	9.258557	7.261675
C	18.879684	10.464521	7.707564
C	19.143091	8.197311	6.556984
C	18.104595	11.458718	8.313610
H	11.760399	13.087744	8.401371
H	9.990689	14.513908	7.363747
H	12.807559	17.730978	6.964504
H	14.587295	16.305502	7.992345
H	15.092993	12.994089	7.430366
H	15.042511	9.999566	8.029184
H	16.436296	8.202051	7.020108
H	19.953184	10.606527	7.569779
H	18.575724	12.364306	8.696483
O	9.072434	16.569441	6.256889
O	10.554871	18.304857	6.219398
O	18.576029	7.091382	6.271621
O	20.332245	8.547869	6.241705
N	17.174496	13.628547	11.549797
N	15.554857	15.324906	11.319801
C	16.804913	14.902150	11.460845
C	15.370148	16.724932	11.206200
C	14.620927	17.425674	12.169340
C	14.395155	18.790939	12.026166
C	14.924074	19.486542	10.923046
C	14.692557	20.936590	10.705044
C	15.708881	18.797344	9.984865
C	15.928101	17.430288	10.124340
C	18.550482	13.387735	11.776828
C	18.965252	12.660085	12.908840
C	20.313358	12.390941	13.117293
C	21.280563	12.852599	12.205087
C	22.729452	12.579065	12.369192
C	20.872240	13.606212	11.092938
C	19.523016	13.866656	10.878186
H	14.229372	16.886669	13.031808
H	13.817090	19.331509	12.775923
H	13.793578	22.434748	11.395674
H	16.126333	19.351288	9.143662
H	16.517893	16.886765	9.385056
H	17.585865	15.666225	11.579263
H	18.219698	12.317752	13.625343
H	20.627275	11.833165	13.999387

H	23.972725	11.663306	13.439610
H	21.631092	13.967703	10.398521
H	19.205549	14.430923	9.999918
O	13.865870	21.491821	11.649010
O	15.162955	21.592607	9.791344
O	22.998564	11.761200	13.438648
O	23.613363	13.008119	11.646421
N	13.005302	12.137096	11.047761
N	13.945484	11.175083	10.992473
C	11.664088	11.833420	10.981449
C	11.228669	10.501398	10.711615
C	9.845698	10.258240	10.609933
C	8.915753	11.277805	10.780985
C	9.355295	12.579071	11.080430
C	12.245516	9.474623	10.580310
C	11.965568	8.122816	10.305868
C	12.977715	7.172557	10.235722
C	14.312742	7.557837	10.451043
C	14.625474	8.878841	10.719153
C	13.607645	9.859179	10.772547
C	10.707626	12.855534	11.183741
H	9.492272	9.250238	10.397605
H	7.850515	11.064423	10.692358
H	8.630212	13.379464	11.234605
H	10.934267	7.810681	10.146326
H	12.734432	6.133031	10.016794
H	15.112106	6.817803	10.397174
H	15.653815	9.189038	10.869937
H	11.057713	13.855760	11.415210
N	15.133881	12.466283	13.249223
N	14.258491	13.500084	13.203954
C	15.463754	11.866291	14.442842
C	14.986843	12.383464	15.687621
C	15.401122	11.761756	16.879330
C	16.241047	10.652344	16.870144
C	16.669170	10.122677	15.641590
C	14.062552	13.502582	15.640895
C	13.479452	14.080026	16.783674
C	12.548030	15.108957	16.686668
C	12.168008	15.576811	15.417371
C	12.726885	15.036028	14.269428
C	13.694708	14.004403	14.353144
C	16.285604	10.712452	14.446619
H	15.050543	12.149090	17.835764
H	16.546389	10.190147	17.809284
H	17.302210	9.234362	15.622590
H	13.751945	13.707469	17.770767
H	12.108248	15.535535	17.588593
H	11.421617	16.367846	15.330218
H	12.421354	15.381789	13.286765
H	16.597715	10.295166	13.494189
